# Two somatic mutations in the androgen receptor N-terminal domain are oncogenic drivers in hepatocellular carcinoma

**DOI:** 10.1038/s42003-023-05704-2

**Published:** 2024-01-05

**Authors:** Qian-Nan Ren, Dan-Hui Huang, Xiao-Nan Zhang, Yue-Ning Wang, Yu-Feng Zhou, Mei-Yin Zhang, Shuo-Cheng Wang, Shi-Juan Mai, De-Hua Wu, Hui-Yun Wang

**Affiliations:** 1 Department of Radiation Oncology, Nanfang Hospital, Southern Medical University, Guangzhou, 510515 Guangdong Province China; 2 State Key Laboratory of Oncology in South China, Collaborative Innovation Center for Cancer Medicine, Sun Yat-Sen University Cancer Center, Guangzhou, Guangdong Province 510060 China; 3 Department of Respiratory and Critical Care Medicine, Chronic Airways Diseases Laboratory, Nanfang Hospital, Southern Medical University, Guangzhou, Guangdong Province China

**Keywords:** Oncogenes, Cancer metabolism, Hepatocellular carcinoma

## Abstract

The androgen receptor (*AR*) plays an important role in male-dominant hepatocellular carcinoma, and specific acquired somatic mutations of *AR* have been observed in HCC patients. Our previous research have established the role of *AR* wild type as one of the key oncogenes in hepatocarcinogenesis. However, the role of hepatic acquired somatic mutations of *AR* remains unknown. In this study, we identify two crucial acquired somatic mutations, *Q62L* and *E81Q*, situated close to the N-terminal activation function domain-1 of *AR*. These mutations lead to constitutive activation of *AR*, both independently and synergistically with androgens, making them potent driver oncogene mutations. Mechanistically, these N-terminal *AR* somatic mutations enhance de novo lipogenesis by activating sterol regulatory element-binding protein-1 and promote glycogen accumulation through glycogen phosphorylase, brain form, thereby disrupting the *AMPK* pathway and contributing to tumorigenesis. Moreover, the *AR* mutations show sensitivity to the *AMPK* activator A769662. Overall, this study establishes the role of these N- terminal hepatic mutations of *AR* as highly malignant oncogenic drivers in hepatocarcinogenesis and highlights their potential as therapeutic targets for patients harboring these somatic mutations.

## Abstract (web summary)

Several mutations proximal to the activation function domain-1 in androgen receptor lead to its constitutive activation and drive the development of hepatocellular carcinoma.

## Introduction

Androgen receptor (*AR*) is a member of the steroid hormone receptor superfamily^1^. Upon stimulation by androgen, *AR* is nuclear translocated and activated to bind with androgen response elements (AREs) in the promoter region of target genes, thereby regulating cell growth and differentiation in males^2^. In the context of tumorigenesis, *AR* plays an important oncogenic role, particularly in sex-biased tumors such as prostate cancer^3^. In prostate cancer, *AR* is an established driver oncogene and therapeutic target^4^. Targeted treatments utilizing *AR* inhibitors, castration, and other endocrine therapies have successfully reduced the risk of death in patients with advanced prostate cancer by approximately 30–40%^3,5^. However, with prolonged treatment, there has revealed various molecular mechanisms leading to castration resistance in prostate cancer (CRPC), including aberrant overexpression of the *AR* gene, persistent activity of splice variants, and, notably, acquired somatic mutations^6^.

Somatic mutations in the *AR* gene have been found to be particularly prevalent in CRPC, where its somatic mutation rate is about 16.8% (72/429) based on data from 429 prostate cancer patients in the Cancer Genome Atlas (TCGA) database. Several studies have proved that somatic missense mutations in *AR* are closely associated with important molecular functions of *AR*, such as transcription activation, protein localization, protein stability, and dimer formation of *AR*, leading to the growth and survival of prostate cancer cell and causing drug resistance to anti-androgen treatment, ultimately resulting in highly malignant castration-resistant prostate cancer (CRPC)^7,8^.

Hepatocellular carcinoma (HCC), another significant sex-biased tumor, exhibits a male-to-female morbidity ratio ranging from 2:1 to 7:1, irrespective of etiology, ethnicity, and geography^9^. Notably, our previous research has demonstrated the role of constitutively active *AR* in hepatocarcinogenesis^10,11^. Specifically, *AR* is a direct target of the driver oncogene mechanistic target of rapamycin complex 1 *(mTORC*1), which promotes cell metabolism and proliferation in vitro and contributes to the development of liver steatosis and HCC in vivo through *AR* phosphorylation^10^. However, early clinical trials investigating anti-androgen and anti-AR therapies in HCC have yielded disappointing results, providing limited clinical benefits for HCC patients^11–13^. Similar to CRPC, specific *AR* gene-related mutations in HCC may play a crucial role in this context. Nonetheless, the functional implications and underlying mechanisms of acquired hepatic *AR* mutations remain unknown.

In this study, we have established the role of two crucial hepatic N-terminal somatic mutations of *AR*, namely *Q62L* and *E81Q*. These mutations result in the continuous hyperactivation of the *AR* protein, leading to the induction of de novo lipogenesis and glycogen accumulation both in vitro and in vivo. Additionally, these mutations suppress the *AMPK* pathway, contributing to hepatocarcinogenesis in vivo. Furthermore, we have observed that hepatoma cells expressing these *AR* mutations exhibit notable sensitivity to the AMPK activator A769662. Thus, this study certifies and emphasizes the value of N-term hepatic mutations of *AR* as highly malignant oncogenic drivers in HCC and identifies their drug sensitization to the AMPK activator, which not only provides further understanding of *AR* in hepatocarcinogenesis but also holds promising potential as a guide for clinical treatment strategies targeting HCC patients with these specific *AR* mutations.

## Results

### Hepatic acquired missense mutations have been observed in HCC patients

**Fig. 1:**
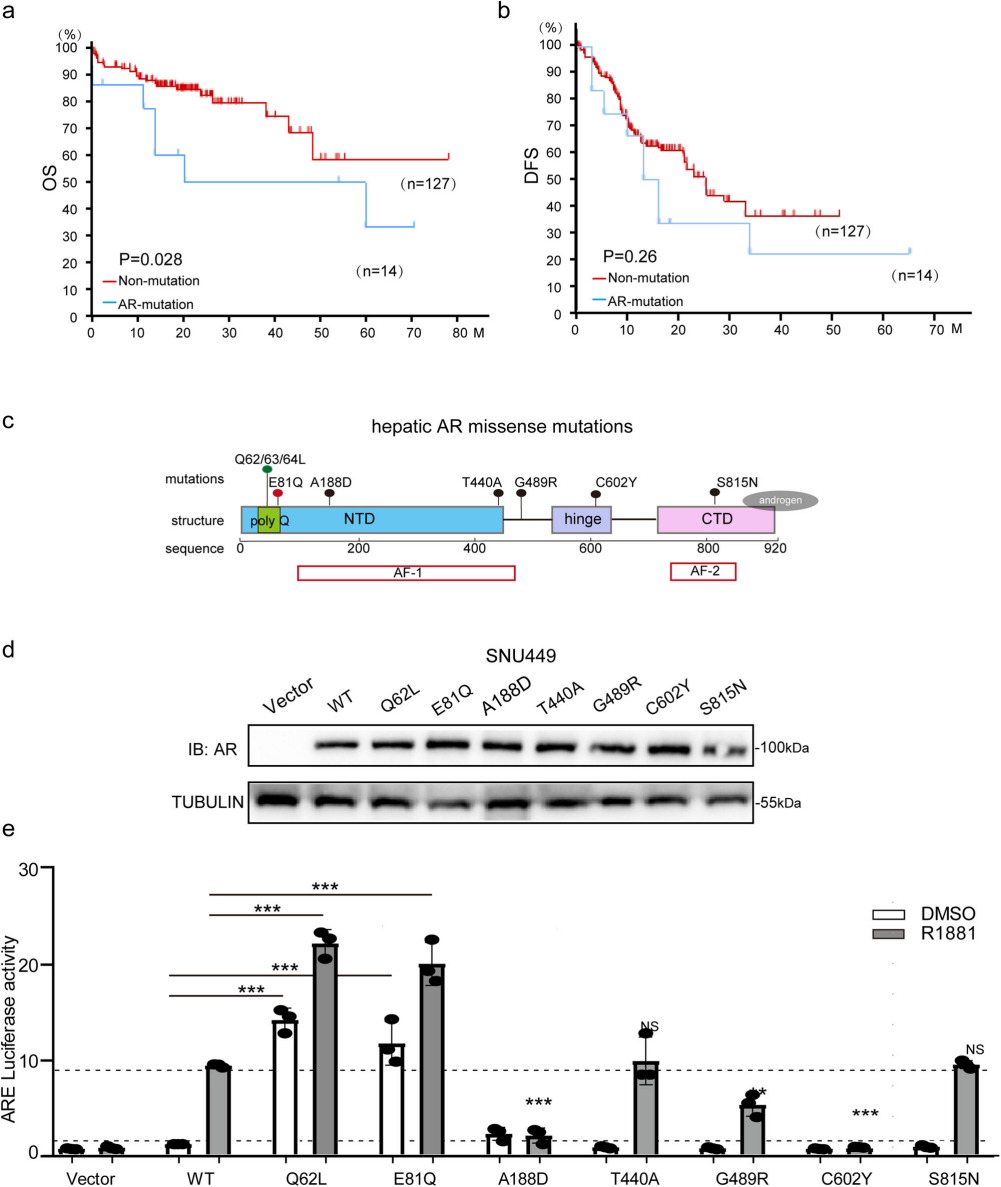
There are two transcription activated mutations close to the polyQ domain of the N-terminal region of *AR*. **a** Patients with HCC and *AR* mutation genes have worse overall survival. Kaplan-Meier analysis and log rank test of OS in patients with AR WT (*n* = 127) versus AR mutations (*n* = 14) expression. **b** Patients with HCC and *AR* mutation genes show no statistically significant of Disease-free survival to patients with HCC and AR WT genes. Kaplan-Meier analysis and log rank test of DFS in patients with AR WT (*n* = 127) versus AR mutations (*n* = 14) expression. **c** There are 7 hepatic missense mutations of *AR* gene in HCC patients. Shown was the sequence and the location of mutations. **d**, **e** Two N-term active somatic mutations (Q62L/E81Q) of AR in HCC patients was found. Shown was the the expression level (**d**) and the transcription activity under ARE control (**e**) of all AR hepatic somatic mutations in SNU449 cells. Protein data was detected by Western Blot and tubulin was analyzed as a control. HA-AR were from different lanes on the same gel (gel# 1). TUNULIN were from different lanes on a separate gel (gel# 2). The molecular weights of HA-AR bands were estimated from the manufacturer guidelines. Luciferase activity was detected by dual-Luciferase reporter assay in the absence or presence of 10^ (-8) M synthetic androgen R1881 for 24 h. Data (mean ± SD, *n* = 3) was analyzed by one-way ANOVA; ***p < 0.001.

To determine if *AR* mutation plays a role in HCC, we analyzed a transcriptome dataset of TCGA database, comprising 366 primary HCC tumor samples. Our analysis revealed an overall acquired somatic missense mutation rate of approximately 3.8% (14/366) in the *AR* gene among HCC patients (http://www.cbioportal.org/)^14^. Remarkably, patients with hepatic *AR* mutations exhibited significantly worse overall survival (OS) compared to those with wild-type *AR* (AR WT), as determined by Kaplan-Meier analysis (*P* = 0.048) (Fig. 1a). Consistently, although there was no statistical difference in progression-free survival (PFS) between the *AR* mutations and *AR* WT groups, a trend towards poorer survival was also observed in the mutation group (Fig. 1b). These findings suggest that somatic *AR* mutations may play an important role in HCC and warrant further investigation.

Among HCC patients, seven types of hepatic *AR* missense mutations have been reported, including *Q62L/Q63L/Q64L* (9/366), *E81Q* (1/366), *A188D* (1/366), *T440A* (1/366), *G489R* (1/366), *C602Y* (1/366), and *S815N* (1/366) (Fig. 1c). Considering the polymorphism of the human polyQ region, it is believed that *Q62L*, *Q63L* and *Q64L* mutations may have similar functions. Therefore, this study focuses on investigating the functional impact of the *Q62L* mutation at these sites. To investigate the role of these mutations in HCC, we constructed plasmids expressing different hepatic *AR* missense mutations (Supplementary Fig. 1). And then, we transiently expressed all these *AR* mutations in SNU449 cells and detected their transcription activity respectively using a luciferase reporter under the control of the androgen response element (ARE) system. Compared with wild-type *AR* (AR^WT^), two mutations located close to the AF-1 domain at the N-terminal region of the AR protein, *AR*^Q62L^ and *AR*^E81Q^, exhibited significantly higher transcriptional activity, both in the presence and absence of androgen. Additionally, two mutations (*AR*^T440A^ and *AR*^S815N^) displayed similar levels of activation, while three mutations (*AR*^A188D^, *AR*^G489R^, and *AR*^C602Y^) demonstrated hyper-suppressive effects (Fig. 1c–e). Considering that *AR* acts as an oncogene in liver diseases^15^, the hyperactivated mutations of *AR* may hold greater effect in HCC. Therefore, we focused on these two hyperactive mutations, *AR*^Q62L^ and *AR*^E81Q^ (Fig. 1c–e). Conclusively, we imply the importance of *AR* gene mutation in HCC and identify two hyperactivated AR somatic mutations in HCC patients.

### Two acquired N-terminal somatic mutations regulate *AR* activity and promote hepatoma cell growth and proliferation

**Fig. 2:**
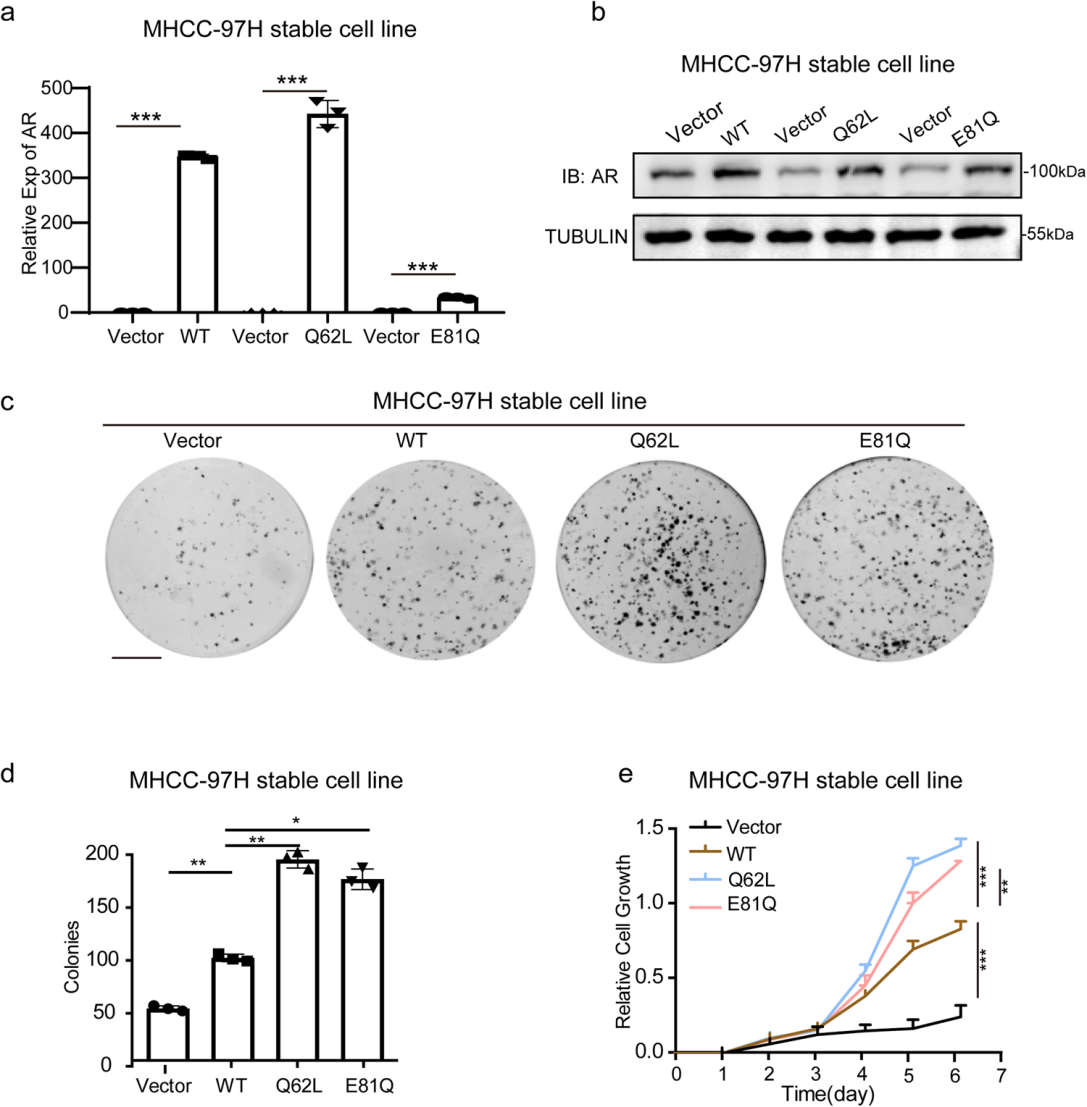
Two activated mutations of *AR, Q62L* and *E81Q* obviously induce the hepatoma cell growth and proliferation. **a**, **b** Shown was the stable expression of *AR WT, AR Q62L* and *E81Q* in MHCC-97H cells. The mRNA level (**a**) and the protein level (**b**) were shown. mRNA data was detected by qRT-PCR. Data (mean ± SD, *n* = 3) was normalized by *GAPDH* and analyzed by one-way ANOVA; ***p < 0.001. Protein data was detected by Western Blot and TUBULIN was analyzed as a control. **c**, **d** Two N-term active somatic mutations *(Q62L/E81Q*) of *AR* induce cell clone formation function in MHCC-97H stable expression cell line. The colonies of 1000 cells of different group after 14 days were shown. Data (mean ± SD, *n* = 3) was analyzed by one-way ANOVA; *p < 0.01; **p < 0.001. (Scale bar = 1 cm*)*. **e** Two N-term active somatic mutations *(Q62L/E81Q)* of *AR* induce cell growth and proliferation function in MHCC-97H stable expression cell line. The cell growth function among 6 days was detected by CCK-8 were shown. Data (mean ± SD, *n* = 3) was analyzed by one-way ANOVA; **p < 0.001; ***p < 0.001.

To investigate the role of the *AR*^Q62L^ and *AR*^E81Q^ mutations in hepatoma cells, we constructed stable expression of *AR*^Q62L^ and *AR*^E81Q^ respectively in HCC-LM3 and MHCC-97H cells and certified the overexpression level of *AR* mutations respectively on mRNA and protein levels (Fig. 2a, b and Supplementary Fig. 2a, b).

To validate the oncogenic functions of these two *AR* active mutations in hepatoma cells, we conducted colony formation and CCK-8 assay in HCC-LM3 and MHCC-97H cells. We observed that stable expression of *AR* WT promoted hepatoma cells growth and colony formation compared to the control vector. And more importantly, the active mutation types, Q62L and E81Q, exhibited a much stronger oncogenic role than *AR* WT (Fig. 2c–e and Supplementary Fig. 2c–e). Among the different types of *AR* gene in our research, *AR*^Q62L^ showed the highest carcinogenic or cancer-promoting activity in hepatoma cells, followed by *AR*^E81Q^, with *AR* WT exhibiting a lower level of activity (Fig. 2c–e and Supplementary Fig. 2c–e). The degree of malignancy correlated positively with the activation of *AR* gene function. Conclusively, we have demonstrated that the two acquired N-terminal somatic mutations of the *AR* gene, *AR*^Q62L^ and *AR*^E81Q^, exhibit continuous hyperactive function and stronger malignancy in vitro.

### *AR* mutations promotes NRAS-dependent hepatocarcinogenesis in vivo

**Fig. 3:**
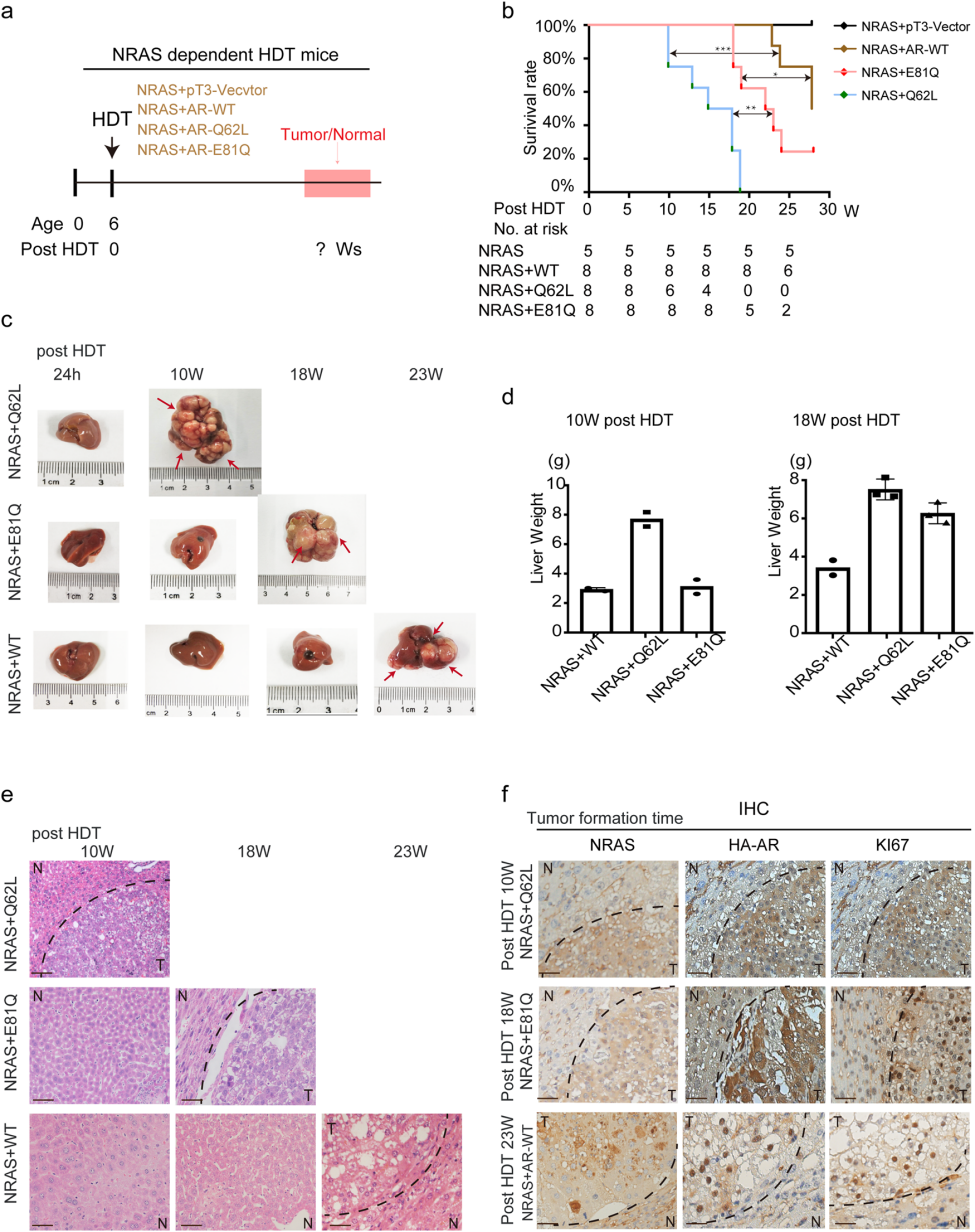
*AR* mutations accelerate hepatocarcinogenesis in vivo. **a** The experimental design of *NRAS/AR*-mutation-driven liver tumorigenesis model in male mice. **b** Kaplan-Meier analysis and log rank test of the survival rates of the mice after HDT injection of NRAS/pT3-Vector (*n* = 5), NRAS/AR-WT (*n* = 8), NRAS/AR-Q62L (*n* = 8) and NRAS/AR-E81Q (*n* = 8). No mortality was observed with the NRAS group during the course of the study. The mean survival of NRAS/AR-WT group was 28 W post HDT, and with AR-Q62L and AR-E81Q, the mean survival of mice was reduced to 15 and 22 W respectively. ***p < 0.001. **c** *AR* mutations accelerate hepatocarcinogenesis of *NRAS*-driven mice compared with WT. The morphological pictures of a representative liver of NRAS/pT3-Vector, NRAS/AR-WT, NRAS/AR-Q62L and NRAS/AR-E81Q HDT mice of different time were shown. **d** The liver weight of different HDT mice at 10 W and 18 W was testes. All error bars represent Standard Deviation. **e** The HE stain of a representative liver of NRAS/pT3-Vector, NRAS/AR-WT, NRAS/AR-Q62L and NRAS/AR-E81Q HDT mice of different time were shown N: normal liver, T: tumor liver (Scale bar = 50 μm). **f** AR mutations have stronger malignancy than WT. The IHC stain of different groups liver cancer at tumor formation time were shown. NRAS/AR-Q62L liver cancer at 10 W post HDT and NRAS/AR-E81Q liver cancer at 18 W post HDT showed higher KI67 stain with 23 W AR-WT mouse. N: normal liver, T: tumor liver (Scale bar = 50 μm).

To assess the oncogenic potential of *AR*^Q62L^ and *AR*^E81Q^ in vivo, we constructed mouse models using hydrodynamic transfection (HDT). *NRAS* alone is insufficient to induce liver cancer in HDT mouse models, but it enhances tumorigenesis when combined with other oncogenes or loss of cancer suppressor genes^16^, Additionally, according to TCGA transcriptome dataset data of HCC patients, there is no expression relationship between *NRAS* and *AR* (Supplementary Fig. 3a), which means that an *NRAS*-accompanying driver HDT mouse model serves as a suitable tool to evaluate the oncogenic events of *AR* mutation genes. Therefore, we evaluated the oncogenic potential of these two *AR* mutations by expressing *NRAS* alone or in combination with HA-AR-Q62L, HA-AR-E81Q and HA-AR-WT respectively in HDT mouse models (Fig. 3a). During the study, *NRAS* alone did not induce any obvious pathological lesions in the mouse liver, as previously reported (Supplementary Fig. 3b)^16^. However, when combined with HA-AR-WT, hepatocarcinogenesis was induced at ~23 weeks post HDT injection. Remarkably, the development of HCC was accelerated to ~10 or ~18 weeks post HDT when *HA-AR*^*Q62L*^ or *HA-AR*^*E81Q*^ were co-injected with *NRAS*, respectively (Fig. 3b). Overexpression of *AR*^*Q62L*^ or *AR*^*E81Q*^ promoted *NRAS*-dependent tumor development, as evidenced by the size of gross tumor nodules and liver weight (Fig. 3c, d). Consistently, the mean survival time for the NRAS/AR^Q62L^ and NRAS/AR^E81Q^ groups was 15 and 22 weeks, respectively, compared with 28 weeks for the NRAS/AR^WT^ group (Fig. 3b). Compared with WT, continuously active *AR*^*Q62L*^ or *AR*^*E81Q*^ mutations in hepatocytes led to faster development of liver tumors in mice. NRAS/AR^WT^ hyper-expressing hepatoma cells were normal at 10~ and 18~ weeks post HDT, and started forming small tumors at 23 weeks. In contrast, livers in the NRAS/AR^E81Q^ group formed preneoplastic lesions by week 10 and showed large tumors lesions by week 18. On the other hand, in the NRAS/AR^Q62L^ group, multiple large tumors already developed by week 10 (Fig. 3e). Consecutive liver sections further confirmed that *AR WT* induce tumor in *NRAS* dependent HDT mice, and the *AR* mutations further accelerated hepatocarcinogenesis as indicated by KI67 staining (Fig. 3f and Supplementary Fig. 3c). Together, these results indicate that *AR*^*Q62L*^ and *AR*^*E81Q*^ mutations activates *NRAS*-driven hepatocarcinogenesis in vivo, highlighting their role as important oncogenic gene mutations in HCC.

### Continuously hyperactivation of *AR-Q62L* or *E81Q* is an independent driver oncogene mutation in hepatocarcinogenesis

**Fig. 4:**
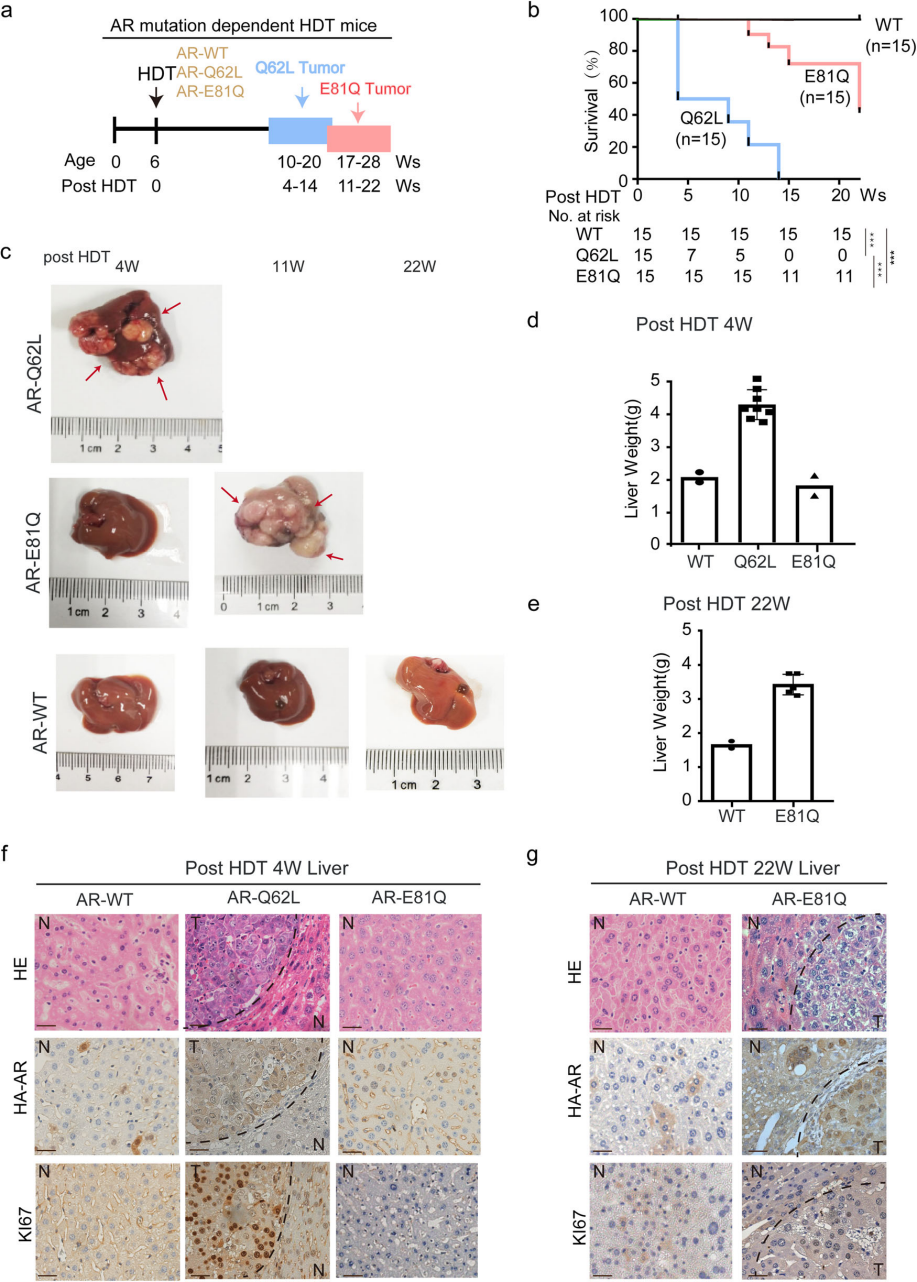
These two activated mutations of *AR* are independent oncogenic mutations to hepatocarcinogenesis. **a** The experimental design of *AR*-mutation-driven HDT mice model. **b** Kaplan-Meier analysis and log rank test of the survival rate and the liver weight of mice after HDT injection of AR-WT (*n* = 15), AR-Q62L (*n* = 15) and AR-E81Q (*n* = 15). The mean survival of AR-Q62L mice was 4 W post HDT, and AR-E81Q mice was 22 W and no mortality was observed in AR-WT mice group. ***p < 0.001. **c** *AR* mutations alone, but not *AR*-WT driver hepatocarcinogenesis in vivo. The morphological picture of a representative liver of the mice models of AR-WT, AR-Q62L and AR-E81Q at different time post HDT. **d** The liver weight of different groups mice at 4 W post HDT was testes.All error bars represent Standard Deviation. **e** The liver weight of different mice at 22 W post HDT was testes. All error bars represent Standard Deviation. **f**, **g** *AR* mutations are independent oncogenic mutations to hepatocarcinogenesis. The HE stains and IHC stains of HA-AR and KI67 among different HDT mice model were shown. N: normal liver, T: tumor liver (Scale bar = 50 μm).

To further determine if *AR* mutations were independent driver oncogenes in hepatocarcinogenesis, we generated liver tumors by HDT-mediated single-gene stable expression of *AR*^Q62L^, *AR*^E81Q^ and *AR*^WT^ (Fig. 4a). In our *AR* mutations driven mice by HDT, we confirmed the similarly transfection efficiency of different *AR* in vitro and in vivo at first (Supplementary Fig. 4a–c). Remarkably, not like *AR*^*WT*^, stable expression of continuously active *AR*^*Q62L*^ or *AR*^*E81Q*^ alone was sufficient to induce hepatocarcinogenesis in mice (Fig. 4b, c). The AR^Q62L^ group mice developed liver cancer starting at 4~ weeks post HDT injection and AR^E81Q^ group mice started to form liver cancer at 11~ weeks. In contrast, livers in the AR^WT^ group were normal all the experiment time (Fig. 4b). At the end of experiment, 22 W post HDT injection, all AR^Q62L^ mice and half of AR^E81Q^ mice presented liver cancer and died. However*, AR*^*WT*^ failed to induce any liver tumorigenesis or caused mortality in mice gross observation throughout the experiment (Fig. 4b, c). Similarly with previous results, *AR*^*Q62L*^*-*expressing mice developed much more robustly as judged by the size of individual tumors than *AR*^*E81Q*^, whereas *AR*^*WT*^ mice did not exhibit apparent tumor and pathological changes (Fig. 4d, e). Histological analysis of the liver tissues further supported these findings. At 4 weeks post HDT injection, AR^Q62L^ liver cancer showed high malignancy, as examined by HE and Ki67 immunohistochemistry, and there were no positive stains or tumors in the AR^E81Q^ or AR^WT^ group. Extending the time to 11~ weeks post HDT injection, AR^E81Q^ liver cancer showed positive KI67 stain and malignancy. On the contrary, AR^WT^ were still negative by week 22, the endpoint of the experiments. (Fig. 4f–g and Supplementary Fig. 4d). These results indicate that *AR* mutations, *Q62L* and *E81Q*, showing higher malignancy than *AR*^*WT*^, and more importantly, are independent driver oncogenic mutations in hepatocarcinogenesis.

To evaluate the malignancy of *AR* mutations in driving HCC, we compared them with other known single-gene drivers of liver cancer, namely *AKT* and *C-MYC*. *AKT* and *C-MYC* signaling are the major oncogenic driver pathways for HCC and hepatic activation of *AKT* or *C-MYC* is sufficient to induce HCC in mouse models through HDT-mediated tail vein injection^17^ (Supplementary Fig. 4e). We found that the survival and tumor burden of *AR*^*Q62L*^ mice and *AR*^*E81Q*^ mice were respectively similar with *AKT* and *C-MYC* HDT mice (Supplementary Fig. 4f). Tissue section analysis at the same time points also showed revealed comparable malignancy between AR^Q62L^ mice and AKT at 4 weeks post HDT, as indicated by KI67 and HE staining. Similarly, AR^E81Q^ mice and C-MYC group mice exhibited similar malignancy at 22 weeks post HDT (Supplementary Fig. 4g). These observations support previous in vitro and in vivo findings, suggesting that unlike the wild-type *AR*, the continuously active mutations *Q62L* and *E81Q* in *AR* are independent driver oncogene mutations in hepatocarcinogenesis and showed high malignancy similar to strong cancer-promoting genes such as *AKT* and *C-MYC*. This comparative analysis reinforces the notion that *AR* mutations play a critical role in driving hepatocellular carcinoma and highlights their oncogenic potential and role in liver cancer development.

### Excessive *AR* mutation promotes liver steatosis, glycogen accumulation in mice

To identify the downstream pathway involved in *AR* mutation-associated hepatocarcinogenesis, we performed chromatin immunoprecipitation sequencing (ChIP-seq) and RNA sequencing (RNA-seq) on tumor tissues and benign liver tissues from AR^Q62L^, AR^E81Q^ and AR^WT^ groups. Through sequencing and bioinformatics analysis, we identified differentially transcriptional or expressed genes between the AR mutation groups and the AR WT group. In ChIP-seq analysis, we found that the transcription binding function of AR was regulated by gene mutation. For example, compared with AR^WT^, there were 641 genes in the AR^Q62L^ group and 2,722 genes in the AR^E81Q^ group which promoter gained the positive binding of *AR*. There were 1,083 genes in the AR^Q62L^ group and 7,285 genes in the AR^E81Q^ group which promoter lost the binding of *AR*. (Supplementary Fig. 5). In RNA-seq analysis, we identified 1,702 upregulated genes in the AR^Q62L^ group and 603 upregulated genes in the AR^E81Q^ group, whereas 6,541 genes were downregulated in the AR^Q62L^ group and 141 genes were downregulated in the AR^E81Q^ group (Supplementary Fig. 5). These findings indicate that AR mutations lead to extensive changes in gene expression profiles, orchestrating a network of downstream genes involved in liver cancer.

**Fig. 5:**
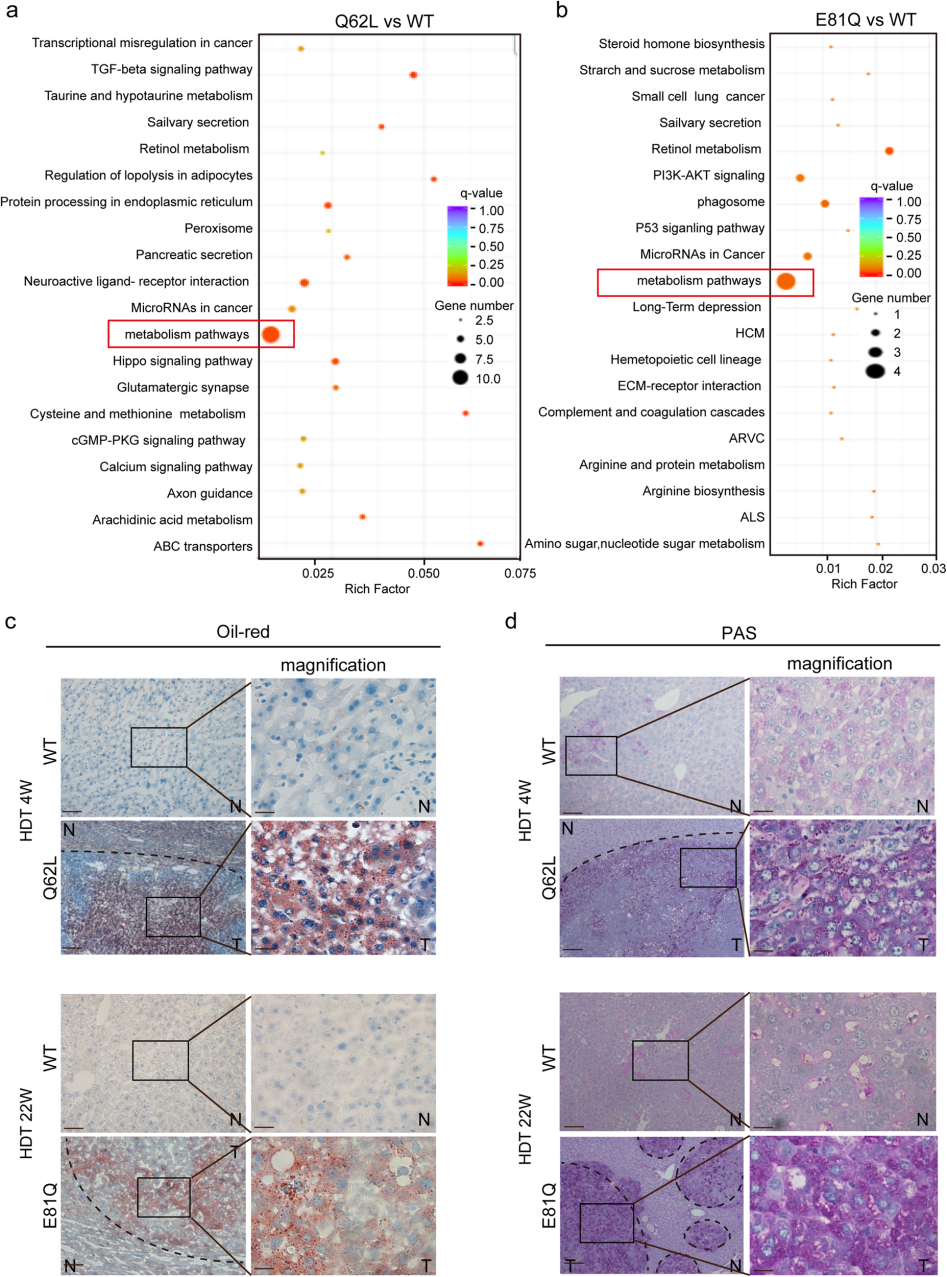
*AR* mutations regulate hepatic metabolic reprogramming. **a**, **b** *AR* mutations are closely related with metabolism pathway. Shown was the KEGG analysis of the cross genes of RNA-sequencing and ChIP-sequencing of *AR*-mutation tumor tissues and AR-WT control tissues. AR-Q62L mutation regulated pathways (**a**) and AR-E81Q mutation regulated pathways (**a**) was shown. **c**, **d** *AR-*mutation activates fat metabolism and glycogen metabolism. Shown was the Oil-red and PAS stain of HDT mice model tissues at different time. N: normal liver, T: tumor liver. (zoomed-out picture, Scale bar = 200μm), (magnifying picture, Scale bar = 50 μm).

Furthermore, when performing KEGG analysis on the differentially expressed genes identified from both ChIP-seq and RNA-seq, we found that the metabolism signaling pathway was specifically enriched and regulated by *AR* mutations in both the Q62L and E81Q groups (Fig. 5a, b), which indicated that *AR* mutations play a role in reprogramming hepatic metabolism during tumorigenesis. In agreement with this, liver tissues from HDT mice expressing *AR*^*Q62L*^ and *AR*^*E81Q*^ showed severe steatosis, as indicated by Oil-Red staining, and prominent glycogen accumulation, as indicated by PAS staining, compared to adjacent tissues and liver tissues from the AR WT group (Fig. 5c, d). Taken together, these results indicated that hepatic acquired activated mutation, *AR*^*Q62L*^ and *AR*^*E81Q*^, act as driver oncogenic mutations in hepatocarcinogenesis by reprogramming liver metabolism, particularly affecting fat and glycogen metabolism.

### Hepatic *AR* acquired mutations activate the transcription of *SREBP1* and *PYGB* to promote abnormal fat and glycogen metabolism to hepatocarcinogenesis

**Fig. 6:**
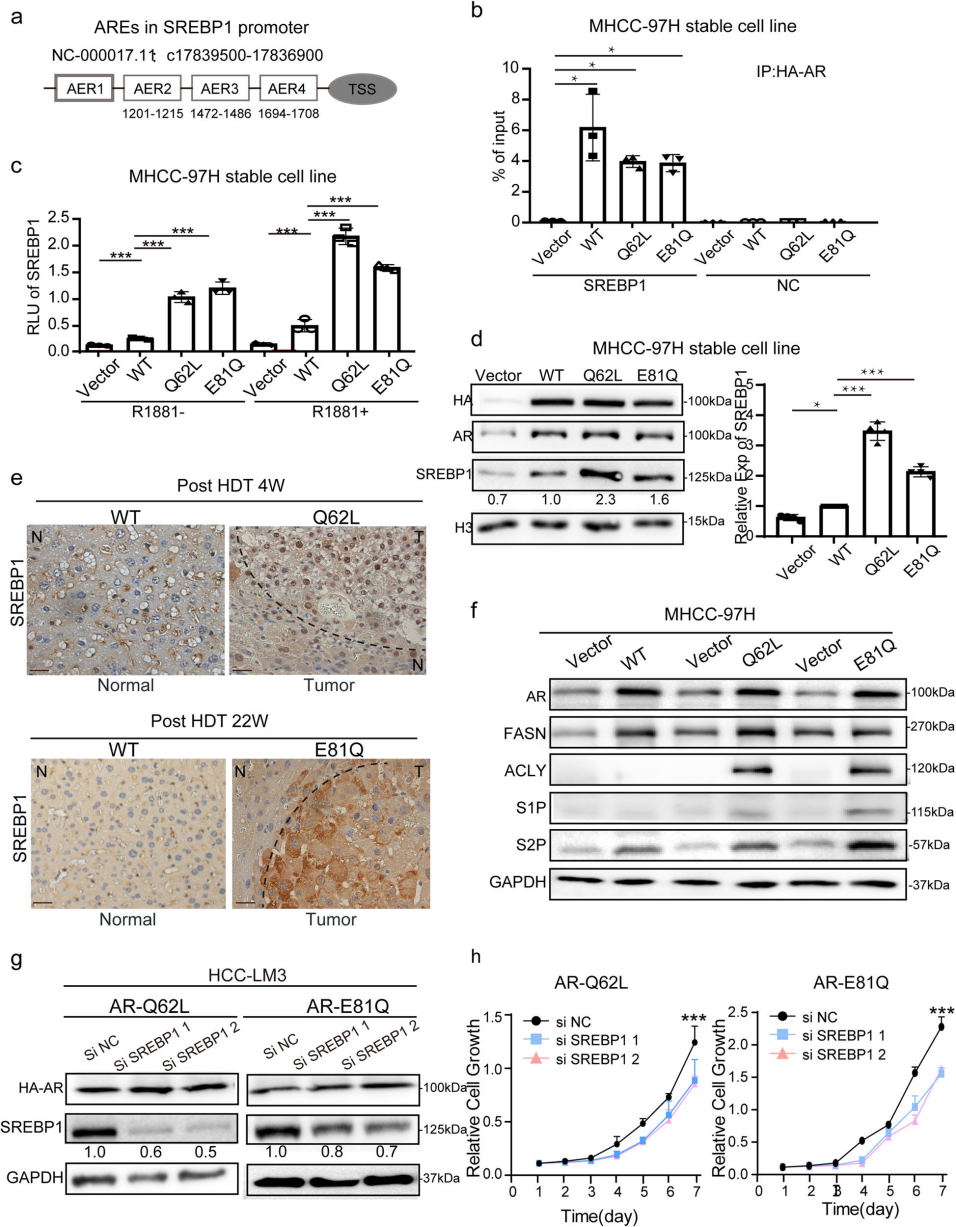
*AR* mutations activated fat metabolism via the SREBP1. **a** Shown are 4 ARE motifs based on the JASPAR ChIP-seq database (http://jaspar.genereg.net/) in SREBP1 promoter. **b** *AR* bond to *SREBP1* promoter. MHCC-97H cells stably expressing HA-AR mutation or HA-AR WT were assayed for *AR* binding to *SREBP1* promoter by ChIP. A random sequence was used as a negative control (NC). Data (Mean ± SD, *n* = 3) was measured by qPCR and analyzed by one-way ANOVA. * p < 0.05. **c** Mutations activated the transcription of *AR* to *SREBP1* promoter. HA-AR WT, HA-AR^Q62L^ and HA-AR^E81Q^ were stably expressed in MHCC-97H cells carrying *SREBP1* promoter-Luc reporter and measured for luciferase activity with or without 24 hours 10^ (-8) M R1881 treatment. Data (Mean ± SD, *n* = 3) was analyzed by one-way ANOVA. ** p < 0.01, *** p < 0.001. **d** *AR* N-term mutations up-regulated the expression of SREBP1 in hepatoma cells. The expression of SREBP1 was detected in MHCC-97H cells by immunoblot and RT-qPCR. H3 and *GAPDH* were used respectively as loading control. The protein level of SREBP1 was examined relative to H3. HA was run on a separate gel (gel #1). AR were from a separate gel (gel #2). H3, SREBP1 were from another gel (gel #3). The molecular weights of H3 bands were estimated from the manufacturer guidelines. The mRNA data (Mean ± SD, *n* = 3) was normalized by *GAPDH* and was analyzed by one-way ANOVA. *p < 0.05, ***p < 0.001. **e** *AR* N-term mutations up-regulated the expression of SREBP1 in HDT mice. The IHC stain of SREBP1 of different HDT mice and control liver tissues were shown. N: normal liver, T: tumor liver (Scale bar = 200/50 μm). **f** *AR* N-term mutations activated the fat metabolism pathway. The expression level of fat metabolism associated genes *(FASN/ACLY/S1P/S2P)* were detected by immunoblot in MHCC-97H cells and GAPDH was as loading control. The molecular weights of GAPDH bands were estimated from the manufacturer guidelines. **g**, **h** *SREBP1* knockdown inhibits the growth and survival of AR-Q62L and AR-E81Q expressing hepatoma cells. MHCC-97L cells stably expressing AR-Q62L and AR-E81Q were treated with *SREBP1*-specific siRNA *(siSREBP1 1/2)* or a control siRNA *(siNC)*. The molecular weights of protein bands were estimated from the manufacturer guidelines. Cell growth was measured by CCK8 assay. Data (mean ± SD, *n* = 3) were analyzed by Repeated measures ANOVA. * p < 0.05, *** p < 0.001.

In the analysis of RNA sequencing data, we identified that the master lipogenic transcription factor *SREBP1* and the key glycogenic rate-limiting enzyme *PYGB* were activated and enriched in liver cancer tissues from both AR^Q62L^ and AR^E81Q^ mice, indicating their involvement in the abnormal fat and glycogen metabolism associated with *AR* mutations in HCC. On the one hand, to investigate the role of AR mutations in *SREBP1*-dependent fatty acid metabolism in HCC, we analyzed the promoter region of *SREBP1* and found four ARE motifs (Fig. 6a and Supplementary Fig. 6a, b). Chromatin immunoprecipitation assays showed that both *HA-AR* WT and the mutations (*Q62L* and *E81Q*) specifically bound to the *SREBP1* promoter. Interestingly, the binding extent was similar between *WT* and mutations (Fig. 6b and Supplementary Fig. 6c). The binding of transcriptional activators *AR* to the *SREBP1* promoter was not significantly affected by different *AR* variants or mutations. Furthermore, we assessed the activity of the *SREBP1* promoter in response to *AR* mutations using a luciferase reporter system controlled by the *SREBP1* promoter respectively in MHCC-97H and HCC-LM3 cells. The results demonstrated that *AR* mutations, regardless of androgen presence, significantly activated *SREBP1* transcription compared to *AR* WT (Fig. 6c and Supplementary Fig. 6d, e). The expression levels of *SREBP1* and associated lipogenic genes (*FASN, ACLY, S1P* and *S2P*) regulated by *AR* mutations were markedly upregulated, both in vitro and in vivo (Fig. 6d–f and Supplementary Fig. 6f–h). Additionally, knockdown of *SREBP1* using siRNA in HCC-LM3 cells with stable expression of *AR*^Q62L^ or *AR*^E81Q^ partially suppressed the growth and survival of hepatoma cells, despite unregulated AR expression (Fig. 6g–h). These observations showed that *AR* mutations act as stronger transcriptional activators of the de novo lipogenic pathway than *AR* WT in driving hepatocarcinogenesis.

**Fig. 7:**
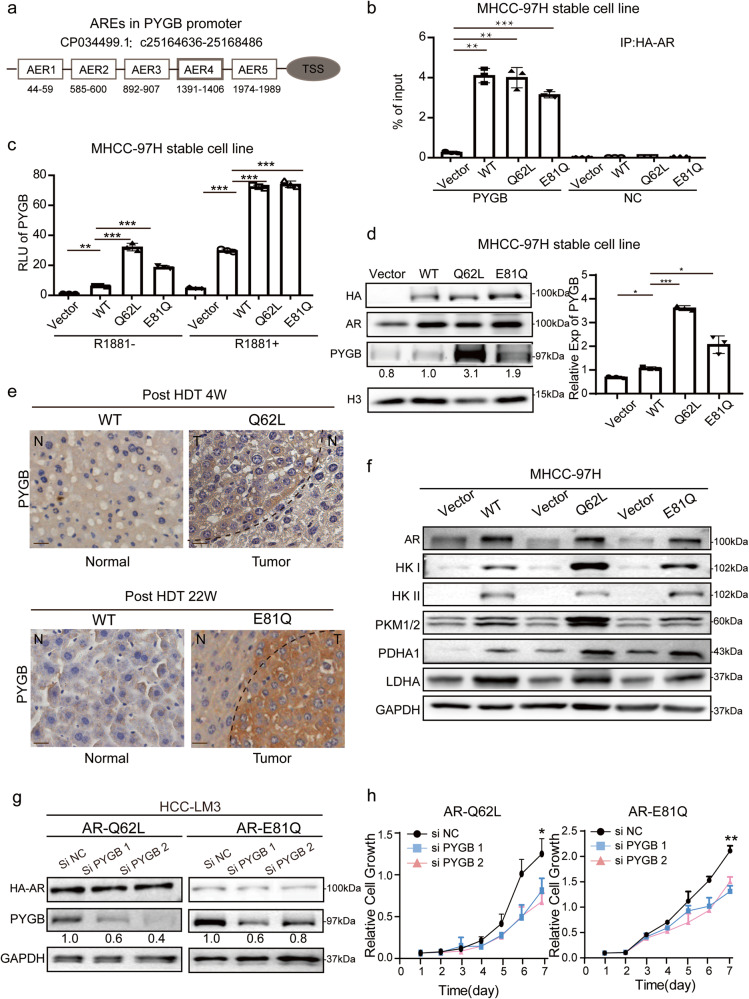
*AR* mutations activated glycogen metabolism via the PYGB. **a** Shown are 5 ARE binding motifs based on the JASPAR ChIP-seq database in PYGB promoter. **b** *AR* bond to *PYGB* promoter. MHCC-97H cells stably expressing HA-AR mutation or HA-AR WT were assayed for HA-AR binding to *PYGB* promoter by ChIP. A random sequence was used as a negative control (NC). Data (Mean ± SD, *n* = 3) was measured by qPCR and analyzed by one-way ANOVA. *p < 0.05, ***p < 0.001. **c** *AR* mutations activated the transcription of *AR* to *PYGB* promoter. HA-AR WT, HA-AR^Q62L^ and HA-AR^E81Q^ were stably expressed in MHCC-97H cells carrying PYGB promoter-Luc reporter and measured for luciferase activity with or without 10^ (-8) M R1881. Data (Mean ± SD, *n* = 3) was analyzed by one-way ANOVA. *p < 0.05, **p < 0.01, ***p < 0.001. **d** *AR* mutations up-regulated the expression of PYGB in vitro. The expression of PYGB was detected in MHCC-97H cells expressing HA-AR WT, HA-AR^Q62L^ and HA-AR^E81Q^ by immunoblot and RT-qPCR. H3 and *GAPDH* were used respectively as loading control. The protein level of PYGB was examined relative to H3. The molecular weights of protein bands were estimated from the manufacturer guidelines. The mRNA data (Mean ± SD, *n* = 3) was normalized by *GAPDH* and analyzed by one-way ANOVA. *p < 0.05, **p < 0.01, *** p < 0.001. **e** *AR* N-term mutations up-regulated the expression of PYGB in HDT mice. The IHC stain of PYGB of different HDT mice and control liver tissues were shown. N: normal liver, T: tumor liver (Scale bar = 50 μm). **f** *AR* N-term mutations activated the glycogen metabolism pathway. The expression level of glycogen metabolism associated genes (*HK1/HK2/PKM1/2/PDHA1/LDHA)* were detected by immunoblot in MHCC-97H cells and GAPDH was a loading control. The molecular weights of some protein bands were estimated from the manufacturer guidelines. **g**, **h** *PYGB* knockdown inhibits the growth of AR-Q62L and AR-E81Q expressing hepatoma cells. MHCC-97L cells stably expressing AR-Q62L and AR-E81Q were treated with *PYGB*-specific siRNA *(siPYGB 1/2)* or a control siRNA *(siNC)*. The molecular weights of some protein bands were estimated from the manufacturer guidelines. Cell growth was measured by CCK8 assay. Data (mean ± SD, *n* = 3) were analyzed by Repeated measures ANOVA. *p < 0.05, **p < 0.01.

On the other hand, to ask the role of *AR* mutations in *PYGB*-dependent glycogen metabolism in HCC, we performed chromatin immunoprecipitation assays and dual-Luc reporter gene assays targeting *PYGB*. The results showed that AR bound to the *PYGB* promoter and regulated its transcription. Importantly, mutations (*Q62L* and *E81Q*) did not influence the binding of *AR* to *PYGB* promoter, but they significantly enhanced the transcriptional activation of *AR* compared to *AR* WT in MHCC-97H and HCC-LM3 cells (Fig. 7a–c and Supplementary Fig. 7a–c). Consistently, *AR*^Q62L^ and *AR*^E81Q^ directly activated the expression of *PYGB* (Figs. 7d, e), accompanying with associated glycogen metabolism genes (*HK1, PKM1/2, PDHA* and *LDHA*) upregulated both in vitro and in vivo (Fig. 7f and Supplementary Fig. 7d–f). Furthermore, when *PYGB* was suppressed using siRNA in HCC-LM3 cells expressing *AR*^Q62L^ and *AR* ^E81Q^, there was a partial inhibition of cell growth and survival (Fig. 7g, h). These results indicate that *AR* mutations are stronger regulator of *PYGB* associated glycogen metabolism pathway in HCC.

Taken together, these findings demonstrate that *AR* mutations activate the transcriptional function of *AR* on both *SREBP1* and *PYGB*, thereby regulating fat and glycogen metabolism in HCC. This suggests that *AR* mutations contribute to the metabolic reprogramming and ultimately promotes the development and progression of liver cancer.

### The suppression of *AMPK* is correlated with hepatic metabolic abnormalities and tumorigenesis

**Fig. 8:**
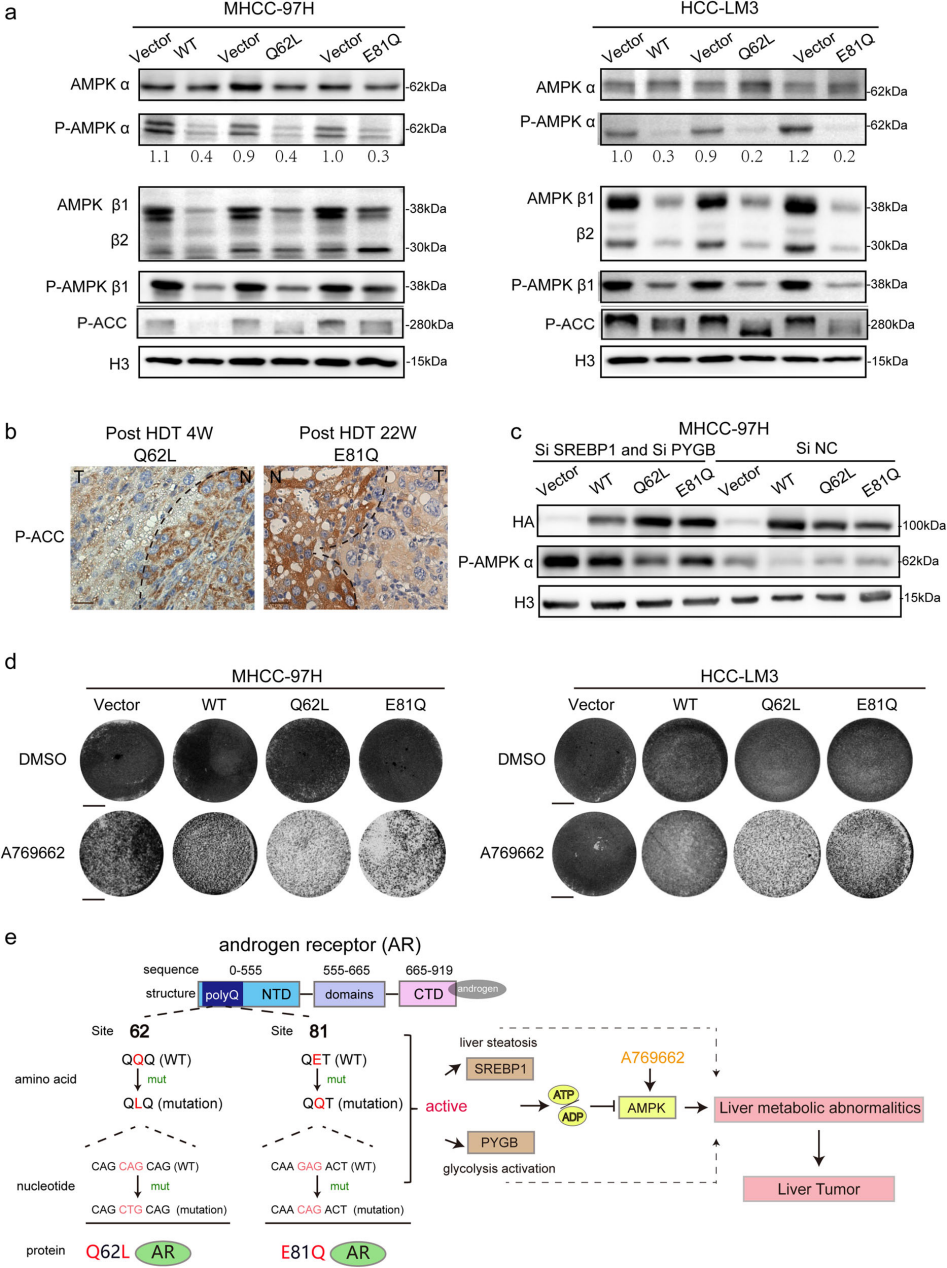
These two *AR* mutations suppress AMPK pathway to tumorigenesis. **a** The activation of *AR* pathway suppressed *AMPK* pathway. MHCC-97H and HCC-LM3 cells stably expressing HA-AR mutations or HA-AR WT were assayed for the expression of AMPK pathway by immunoblot. The protein level of P-AMPK ɑ was normalized to total AMPK ɑ and H3 was used as a loading control. **b** Two N-term mutations of *AR* suppressed P-ACC expression in vivo. P-ACC is the direct gene of AMPK pathway. Shown was the IHC stain of P-ACC stain of HDT mice tumor tissues of AR-Q62L and AR-E81Q. N: normal liver, T: tumor liver (Scale bar = 50 μm). **c** The suppression of SREBP1 and PYGB activated AMPK pathway. The expression of P-AMPK alpha was shown by immunoblot in SREBP1 and PYGB knock-down cells. H3 was used as a loading control. The molecular weights of some protein bands were estimated from the manufacturer guidelines. **d** AMPK agonist A769662 suppressed cell survival rate of *AR* mutations in hepatocytes. The hepatoma cells expressing AR mutations were dealt with 200uM A769662 or DMSO for 48 h and the survival function were shown by crystal violet staining. (Scale bar = 1 cm). **e** Graphic illustration of AR mutations, Q62L and E81Q regulation of metabolic reprogramming to drive hepatocarcinogenesis.

Considering the disappointing outcomes of anti-androgen and anti-AR therapies in HCC, which have provided limited clinical benefits for HCC patients, our focus primarily lies on exploring inhibitors targeting downstream target genes to address anti-*AR* mutations in HCC. 5’-AMP-activated protein kinase (*AMPK*), an energy sensor and key metabolic regulator that controls energy metabolism including fat and glycogen, is obviously repressed in NASH and HCC^18^. The activated metabolic pathways in liver cancer promote tumorigenesis, in part, by suppressing *AMPK*-dependent metabolic reprogramming^19,20^. Therefore, we hypothesized that *AMPK* might be the key link between *AR* mutation-induced liver metabolic abnormalities and tumorigenesis. To test this hypothesis, we performed western blot analysis in *AR*-expressing MHCC-97H and LM3-HCC cells and found that the activation of *AR*, including both wild-type and mutant forms, significantly downregulated the phosphorylation of *AMPK* alpha and beta subunits (Fig. 8a). Similarly, in the liver tumors of *AR* mutation HDT mice, *AMPK* activity was also markedly suppressed compared to normal liver tissues, as evidenced by the decreased phosphorylation of the *AMPK* target gene, *ACC* (Fig. 8b). To further investigate the role of *AMPK* in AR mutation-driven hepatocarcinogenesis, we suppressed the expression of *PYGB* and *SREBP1*, two key downstream targets of AR mutations, using *siRNA* in hepatoma cells. Interestingly, the suppression of PYGB and SREBP1 resulted in a partial upregulation of the *AMPK* pathway (Fig. 8c). These findings suggest that *AR*^*Q62L*^ and *AR*^*E81Q*^ mutations suppress the *AMPK* pathway in response to the metabolic stimulation induced by these mutations in fat and glycogen metabolism. Next, we tested the effects of *AMPK* activation on cell survival and growth in *AR*-activated MHCC-97H and HCC-LM3 cells using a small-molecule activator called A7269662. The results showed that the activation of *AMPK* by A7269662 significantly inhibited cell viability and growth. Importantly, A7269662 demonstrated greater efficacy in inducing cytotoxicity in hepatoma cells expressing *AR* mutations compared to those expressing wild-type AR (Fig. 8d and Supplementary Fig. 8a). These findings suggest that the *AR* mutation status ultimately leads to the suppression of the *AMPK* pathway, contributing to hepatocarcinogenesis, and also highlight the potential of *AMPK* activators as a treatment strategy for patients with these specific somatic mutations.

## Discussion

Epidemiological studies have consistently shown that men are more prone to developing non-alcoholic fatty liver disease (NAFLD) and hepatocellular carcinoma (HCC) compared to women, regardless of the underlying cause and geographical location^21,22^. Mechanistic studies have primarily focused on the wild-type AR gene and its involvement in HBV-dependent and obesity-associated hepatocarcinogenesis^10,23^. However, whether and how AR gene mutations participated in hepatocarcinogenesis remain poorly understood. This study identified two specific hepatic somatic mutations of the *AR* gene, *AR-Q62L* and *AR-E81Q*, which were found to robustly activate *AR* transcription independent of androgen stimulation. Importantly, these *AR* mutations exhibited stronger malignancy and were capable of driving hepatocarcinogenesis alone in a mouse model (Fig. 8e).

The AR protein consists of the N-terminal domain (NTD), the middle DNA-binding domain (DBD), and the C-terminal ligand-binding domain (LBD)^24^. As a critical transcription activator, it contains two activation domains to regulate its function, respectively located in NTD and CTD. One is androgen dependent and the other is independent, with each of the two activation domains offering ~50% to the total *AR* activity. Among NTD, there has a poly-glutamine (Q) repeats, which are common features of transcription factors, playing the vital role in correct protein folding and structure of the *AR*-*NTD*^25^. Interestingly, among all hepatic missense mutation types, only *AR-Q62l* and *AR-E81Q* mutations are located within or next to the polyQ repeats domain in the NTD, suggesting that these mutations may alter the structure and activate the function of *AR-NTD*. This could explain the continuous and high activation of these mutations, independent of androgen levels. Otherwise, compared with WT gene, even though the fluctuation of hormone level in vivo posed some impact to the activity in *AR* mutations, the function activity is still maintained in a persistently high level. In tumorigenesis, both sustained and high activation of oncogene is of crucial importance, which may explain why the mutations, rather than the wild-type *AR* gene, were sufficient to driver HCC occurrence and development. These findings shed light on the role of *AR* gene mutations in hepatocarcinogenesis and provide insights into the underlying oncogenic mechanisms.

Nutrients provide the building blocks for generating biomass and energy to fuel biochemical reactions. They also act as chemical signals that dictate cellular growth and metabolism. Overnutrition creates imbalanced metabolism and excessive nutrient signaling that promotes aberrant cellular metabolism and proliferation, leading to obesity, NAFLD, and cancer^26,27^. The liver, as the central organ responsible for nutrient processing, is particularly vulnerable to hyperactive nutrient signaling, leading to the development of NAFLD and liver diseases such as HCC^28^. In this study, we found that chronic activation of *AR* mutation signal in liver directly contributes to metabolic abnormalities, specifically in fat and glycogen metabolism, through the dysregulation of key molecules like *SREBP1* and *PYGB*, respectively. Furthermore, abnormal nutrient metabolism pathways eventually lead to the suppression of the *AMPK* pathway, which is known to contribute to the development of HCC. Treatment with an *AMPK* agonist showed promising results in reversing the malignancy and progression of hepatoma cells expressing *AR* mutations (Fig. 8e). These findings highlight the role of *AR* mutations in disrupting normal nutrient metabolism pathways in the hepatocarcinogenesis. In summary, this study reveals the role of two N-terminal *AR* mutations in hepatocarcinogenesis and their potential treatment target. It not only provides insights about the *AR* gene mutations in HCC but also shows potential diagnostic and therapeutic strategy for liver cancer.

However, there are limitations in our study. Firstly, due to technical and financial constraints, we were unable to use *AR* negative models ideally. Instead, we utilized HDT mice models to mimic *AR* mutations in vivo, resulting in a mixture of endogenous *AR* with ectopic *AR* (with and without the mutations) in our data. Despite the significantly high expression of exogenous *AR* observed both in vivo and in vitro experiments, it is crucial to acknowledge that the interference from endogenous *AR* cannot be completely ignored. This may introduce limitations and deviations into our data analysis. Secondly, all the conducted in vivo experiments were performed exclusively on male mice. Further investigation is required to ascertain if these conclusions hold true for females. Finally, in this study, we only provided preliminary evidence suggesting the potential of a small-molecule *AMPK* activator as a therapeutic option for tumors harboring specific mutations in *AR*. The obtained results were insufficient and further investigation is warranted.

## Methods

### HDT animal experiments

#### Mechanism

HDT is a method for long-term gene expression only in mouse hepatocytes. As reported, hydrodynamic transfection uses a hydrodynamic force produced by the pressurized injection of a large volume of DNA solution into the blood vessel, which permeabilizes the capillary endothelium and generates pores in the plasma membrane of the surrounding parenchyma cells. DNA has access to the intracellular compartment through these pores. Subsequently, the pores of the plasma membrane close, trapping the DNA inside the parenchymal cells. The injection of such a large volume of DNA solution entering directly into the inferior vena cava stretches myocardial fibers over the optimal length for contraction, induces cardiac congestion, and drives the injected solution into the liver in retrograde.

#### Methods

Six-week-old uncastrated FVB/n male mice were ordered from Beijing Vital River Laboratory Animal center (Beijing, China), using with the approval of the Laboratory Animal Ethics Committee of Sun Yat-Sen University. HA-AR-WT plasmids was constructed by PCR cloning of AR sequence from pCDH-AR-WT into pT3-Vector plasmid using In-Fusion HD Cloning Kit (Takara #639648). HA-AR-Q62L, HA-AR-E81Q plasmids were generated by PCR-directed mutagenesis using the specific forward (F) and reverse (R) primers shown in supporting information. Other plasmids pT3-myr-AKT-HA (Cat No 31789), Nras v12-caggs (Cat No 20205), c-myc-PT3EF1a (Cat No 92046) and pCMV/SB (sleeping beauty,) were gifts from Prof. X.F. Steven Zheng (RU), and the sequences of all the plasmids were confirmed by Sanger sequencing (Ruibiotech).

##### NRAS+pT3-Vector/NRAS + AR-WT/NRAS + AR-Q62L/NRAS + AR-E81Q HDT mice

10 ug Nras v12-caggs plasmids combining with 2.5 ug sleeping beauty+40ug pT3-Vector/AR-WT/AR-Q62L/AR-E81Q were diluted in 2 ml 0.9% NaCl and were rapidly injected from tail into mice in 5–8 seconds, also known as hydrodynamic injection. Then the mice were observed at least once a week. In order to make a more intuitive comparison between different groups of mice, when one group started to form tumors, two tumor-free mice of other control groups were also sacrificed for comparative observation simultaneously. The deaths resulting from tumor formation were defined as the real endpoints and were recorded as positive. The deaths resulting from artificial sacrifice were not considered as the real end point and weren’t counted in Kaplan Meier curves.

##### AR-WT/AR-Q62L/AR-E81Q HDT mice

40ug pT3-HA-AR-WT/pT3-HA-AR-Q62L/pT3-HA-AR-E81Q plasmids combining with 2 ug sleeping beauty were diluted in 0.9% NaCl and were rapidly injected from tail into mice within 5–8 seconds. The mice were sacrificed at different time depending on experiment and the liver tissues were collected and analyzed. In order to make a more intuitive comparison between different groups of mice, when one group started to form tumors, two tumor-free mice of other control groups were also sacrificed for comparative observation simultaneously. The deaths resulting from tumor formation were defined as the real endpoints and were recorded as positive. The deaths resulting from artificial sacrifice were not considered as the real end point and weren’t counted in Kaplan Meier curves.

##### C-MYC/AKT HDT mice

40ug C-MYC-PT3EF1a/pT3-myr-AKT-HA plasmids combining with 2ug sleeping beauty were diluted in 0.9% NaCl and were rapidly injected from tail into mice in 5–8 seconds as control group.

### Gene correlation analysis, ChIP-seq and RNA-seq analysis

372 HCC patients’ data was collected from TCGA database. R language (version 3.53, Missouri, USA) packages (Gmisc and boot) were used to analyze the expression correlation between AR and NRAS. R (Pearson correlation coefficient) and p value was calculated by R studio.

HA-AR-Q62L and HA-AR-E81Q HDT mice with apparent tumor burden and HA-AR-WT mice with benign liver were sacrificed at different experimental time and the liver tissues were collected immediately and quick-freeze into liquid nitrogen. Then part of all tissues was used for extraction of RNA and RNA-seq in Novogene Bioinformatic Technology (Beijing, China). Another part of tissues was used for ChIP assay with anti-HA antibodies and ChIP-seq in Novogene Bioinformatics Technology (Beijing, China). Anti-IgG antibodies were used as negative control and no addition androgen stimulation or suppression was treated in ChIP assay. Then the differential genes were analyzed and compared by Kyoto Encyclopedia of Genes and Genomes (KEGG) analysis.

### Mutations analysis of AR in HCC patients

The somatic mutations of *AR* in hepatoma patients were collected from Cbioportal database. The data were numbered as ANDR-HUMAN ENST00000374690. In this database, there has identified 14 HCC patients (14/366) with 7 AR missense somatic mutations. All the identified hepatic mutations of AR are including Q62L/Q63L/Q64L, E81Q, A188D, T440A, G489R, C602Y and S815N (http://www.cbioportal.org). Informed consent was obtained from all participants and all ethical regulations relevant to human research participants were followed.

### Cell lines

The human HCC cell lines MHCC-97H, HCC-LM3 and HEK-293T cells were preserved in the State Key Lab of Oncology in South China, and cultured at 37 °C in Dulbecco’s modified Eagle’s medium (Gibco, USA) plus with 10% fetal bovine serum (Invitrogen, USA), 100 units/ml penicillin, and 100 μg/ml streptomycin in a humidified atmosphere containing 5% CO_2_. SNU449 cells were preserved in the State Key Lab of Oncology in South China, and cultured at 37 °C in 1640 medium (Gibco, USA) plus with 10% fetal bovine serum (Invitrogen, USA), 100 units/ml penicillin, and 100 μg/ml streptomycin in a humidified atmosphere containing 5% CO_2_.

All cell lines were gifts from Prof. X.F. Steven Zheng (RU). All cell lines were authenticated by STR DNA profiling (Microread Diagnostics Co., Ltd, Guangzhou, China) and tested for *Mycoplasma* contamination by RT-PCR in our lab.

### Plasmid construction

HA-ARQ62L(Addgene ID 213032), HA-ARE81Q(Addgene ID 213033), HA-ARA188D(Addgene ID213608), HA-ART440A(Addgene ID 213609), HA-ARG489R(Addgene ID 213610), HA-ARC602Y(Addgene ID 213611) and HA-ARS815N (Addgene ID 213612) plasmids were generated by PCR-directed mutagenesis (Initial denaturation 98 °C 10 min; denature 98 °C 5Sec, anneal 60 °C 15Sec, extend 72 °C 1 min/kb (15-20cycle); Final extend 10 min) using the specific following forward (F) and reverse (R) primers. After PCR, the reaction solution was treated with DPNI (NEB #R0176S) for 37 °C 1 h to clear the template AR-WT plasmids. All the plasmids were confirmed by Sanger sequencing (Ruibiotech) and stored in SYSUCC plasmids library (contact corresponding author for new plasmids) and addgene (https://www.addgene.org). The primers sequence was showed in Supplementary Table 4. The plasmids were showed in Supplementary Table 6.

### Construction of stable cell lines

The full-length sequence of *AR-WT* or *AR* mutations was amplified and cloned into the multiple cloning sites of pCDH-CMV-EF1AH, then subcloned into lentivirus to overexpress AR through transfection with psPAX2 (https://www.addgene.org/12260/) and pMD2.G (https://www.addgene.org/12259/) in HEK-293T cells, respectively. Following a 48-h period of infection with lentivirus plus 5 mg/ml Polybrene, stable cells with expression of AR were selected with 4 μg/mL puromycin for 1 week. After selection, the cells were continued with medium containing 2 μg/mL puromycin.

### Double luciferase reporter gene experiment

#### ARE luciferase report assay

1 × 10^5 cells were seeded in 24-well (NEST) plates and then transfected with 1ug pGMARE-Lu and 10 ng pRL-TK for 24 h. The double luciferase reporter gene was determined using the dual-luciferase report assay kit (Promega) and was tested by GloMax2020 Luminescence detector E5331 (Promega) according manufacturer’s instructions. The plasmids pGMARE-Lu were purchased from Shanghai YEASEN company (https://www.yeasen.com/products/detail/1296) and were confirmed in our previous studies.

#### SREBP1 luciferase report assay

PGL4.10-SREBP1 reporter plasmids were constructed by PCR cloning of SREBP1 promoter into PGL4.10-Basic plasmids using the In-Fusion HD Cloning Kit (Takara #639648) and were confirmed by Sanger T

#### PYGB luciferase report assay

PGL4.10-PYGB reporter plasmids were constructed by PCR cloning of PYGB promoter into PGL4.10-Basic plasmids. Then the double luciferase reporter gene assay was performed as before. There were at least three additional holes in each experiment and three independent experiments were conducted.

### Cell proliferation assays

Cell proliferation was assessed by CCK-8 Cell Counting Kit (Dojindo Laboratory, Kyushu, Japan) and colony formation assay. For CCK-8 assay, cells were seeded into 96-well plates at a density of 1000 cells per well and incubated for 5-6 days with different treatment under 5% CO_2_. Cells were treated with CCK-8 solution for 1 hours and the growth rate of cells was determined by absorbance at 450 nm with SpectraMax M5 Multi-Mode Microplate Reader (Molecular Devices LLC, Sunnyvale, CA, USA). For cell killing assay, cells were seeded into 6-well plates (10^5 cells per well) and cultured with different treatment for 48 h, then fixed by methanol for 10 minutes and stained with 2% crystal violet solution (Beyotime) for 2 hours. Images of Colonies were captured by ChemiDoc Imaging Systems (Bio-Rad, California, USA) and the number of colonies were counted by Image J software.

### IHC

The IHC was carried out as protocol described. The Antigen retrieval was conducted by pressure cooker at full pressure for 3 min in the EDTA Buffer pH=8.0 (ORIGENE # ZLI-9067) and then blocked in 10% FBS for 1 hour. Then the paraffin sections were incubated with the first antibodies diluted in antibodies diluent buffer (ORIGENE # ZLI-9029) at 4 °C overnight and with Horseradish Peroxidase (HRP)-conjugated secondary antibody (ORIGENE # ZLI-9017) at 37 °C for 1 h. Finally, the DAB chromogenic solutions (ORIGENE # ZLI-9017) were used to detect the positive staining. In the IHC assay of this study, at least three liver tissues of each tumor-forming mice group and at least two liver tissues of each control mice group were collected for IHC staining, and at least three to five photos with different views were took to confirm the staining result.

### Western blot analysis

Protein extracts were prepared using RIPA buffer with protease inhibitor cocktail (Ape Bio). 20 ug of protein extract were run per well on precast SDS–PAGE gels (Bio-Rad) and transferred to 0.45 PVDF membranes prior to imaging with the Bio-Rad ChemiDoc Imaging System.

In Figs. 1d and 6d, 20 ug of the same protein samples were used in different gels for western blot analysis. The Protein bands for Figs. 1d and 6d were not derived from the same original blot and the details were shown in figure legends. The molecular weights of some protein bands were estimated from the manufacturer guidelines, rather than verified with a molecular weight ladder.

### Antibodies, Primers and SiRNA

The antibodies and qPCR-primers used were showed in Supplementary Table 1 and Supplementary Table 2. The siRNA was showed in Supplementary Table 5.

### ChIP

ChIP was performed using a Millipore EZ-ChIP kit (catalog#17-371). Cells in a 10-cm dish (BIOFIL) were fixed with 1% formaldehyde for 10 min, stopped fixing with 0.125 M Glycine (MDbio). According to the protocols, the chromatin was subjected to sonication to 100–500 bp and then were incubated with anti-HA antibodies (Abcam) for overnight at 4 °C using automatic epigenetic sample processing system (diagenode IP-Star®). The qPCR-primers used were showed in Supplementary Table 3.

### Statistics and Reproducibility

The number of data used in the analyses (n) is presented alongside the corresponding p-value. For experiments in Figs. 3d, 4d, e, Supplementary Fig. 6h and Supplementary Fig. 7h, we conducted two independent replicates for control groups. For other experiments, we conducted a minimum of three independent replicates for each group. The complete description of replicate numbers (and the corresponding data numbers used for analysis) at each sampling time can be found in the Figure legends. In our study, we calculated both the average value and standard deviation of the data. To compare differences between data groups, one-way ANOVA tests were performed.

### Supplementary information

Supplementary Information

Description of Additional Supplementary Files

Supplementary Data 1

reporting-summary

## Data Availability

Data supporting the findings of this work are available within the paper and in the Supplementary files. All uncropped blots for Figs. 1–8 and Supplementary Figs. 1–8 is included in Supplementary Fig. 9. The source data behind the graphs and charts is available in Supplementary Data 1. Source data of sequencing is stored in GEO database (RNA-seq: GSE249685, ChIP-seq: GSE249686). All newly generated plasmids were stored in addgene (Supplementary Table 6). The data sets for this study are available from the corresponding author upon request.
