# Supplementary material for: Two somatic mutations in the androgen receptor N-terminal domain are oncogenic drivers in hepatocellular carcinoma
Source: Commun Biol. 2024 Jan 5;7:22. doi: 10.1038/s42003-023-05704-2 (PMC10770045; doi:10.1038/s42003-023-05704-2)
Supplement: Supplementary file 1 — Supplementary Information [file 42003_2023_5704_MOESM1_ESM.pdf]

Supporting Information

1. Supplementary Figure 1-9

2. Supplementary Table 1-6

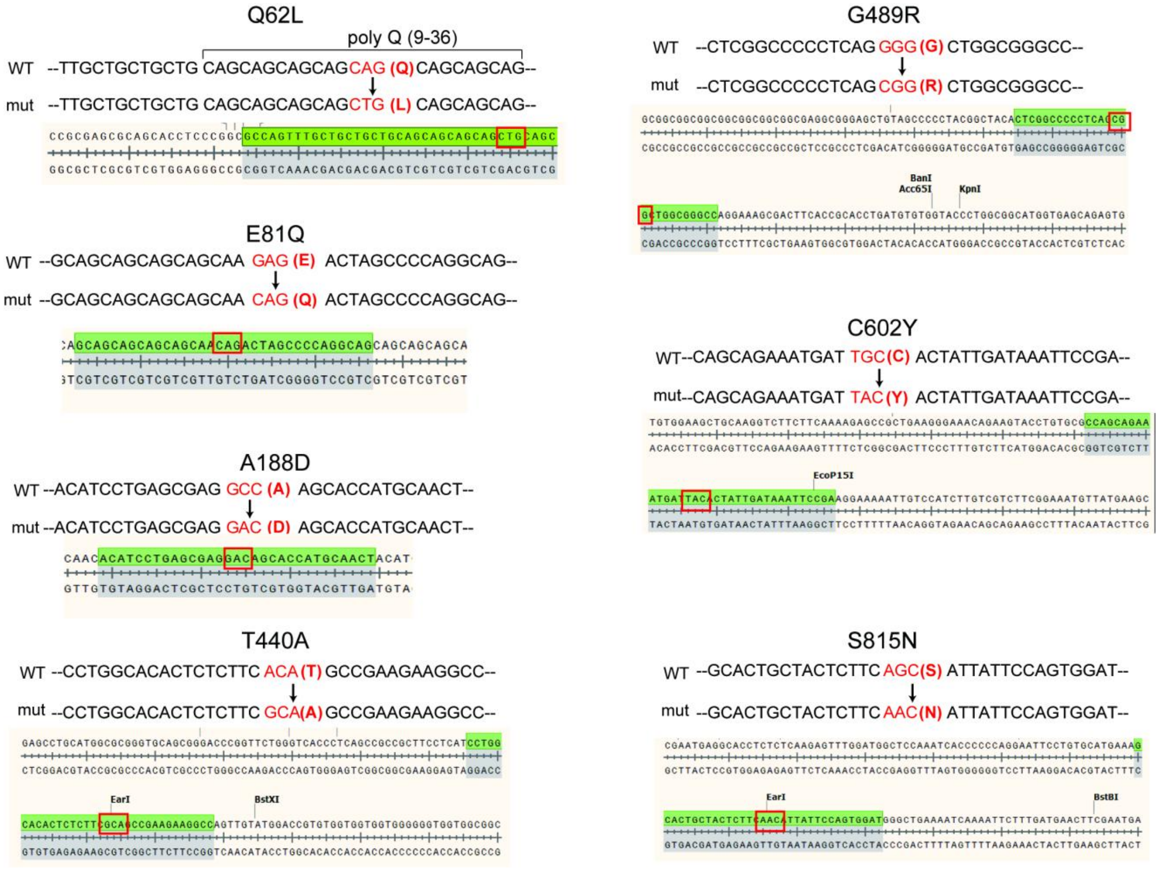

Supplementary Figure 1. The sequence of hepatic AR missense mutations.

Shown was the sequence of 7 AR missense mutations of HCC patients in plasmids.

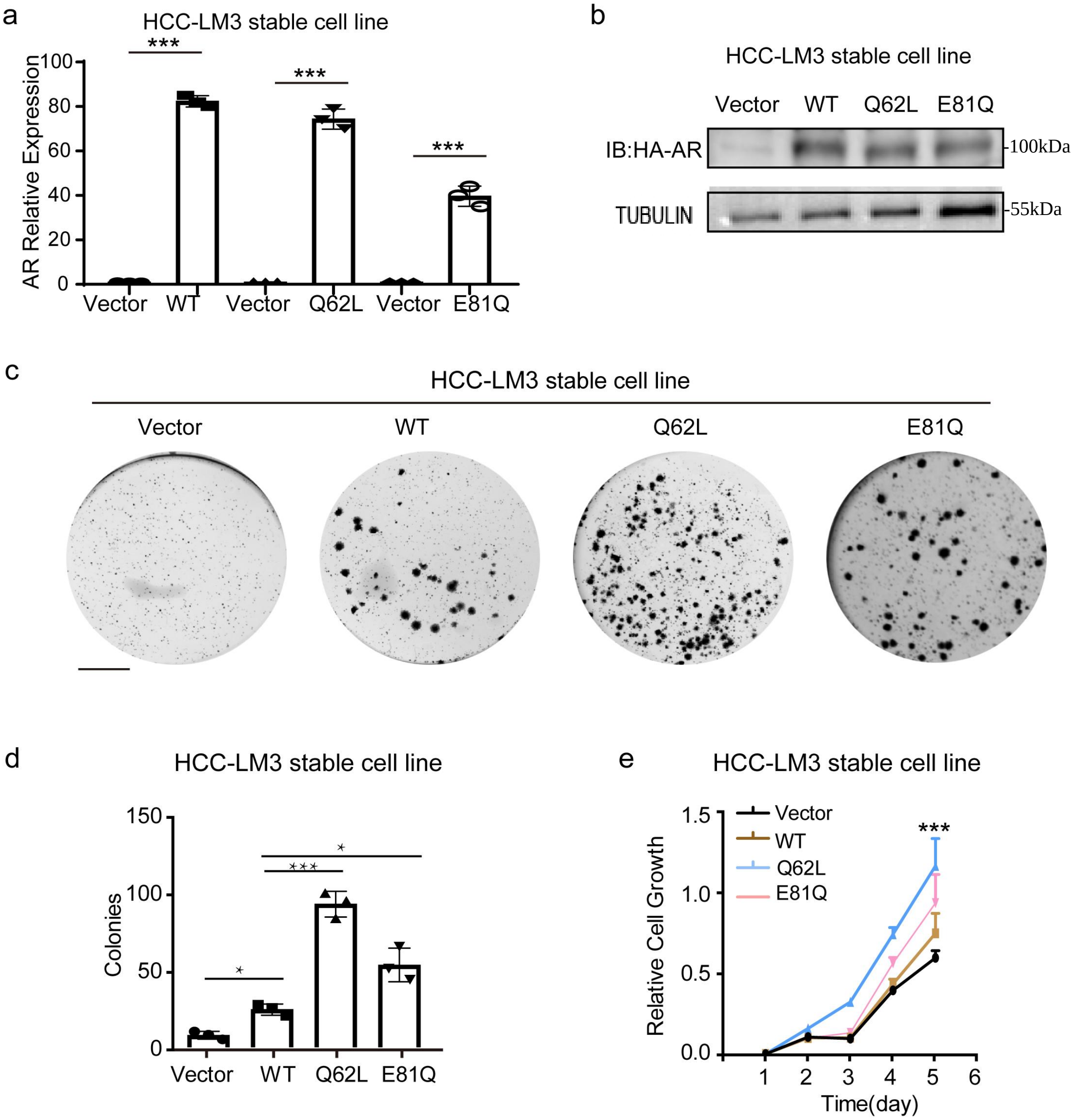

Supplementary Figure 2. AR mutations induced the hepatoma cell growth and proliferation than wild-type in HCC-LM3 cell.

a-b. Shown was the stable expression of AR WT and AR mutations, Q62L and E81Q in HCC-LM3 cells. The mRNA level (a) and the protein level (b) were shown. mRNA data was detected by qRT-PCR and data (mean±SD， *n*=3) was

analyzed by one-way ANOVA; ****p<0.001.* Protein data was detected by Western blot and TUBLIN was analyzed as a control control.

c-e. Two N-term active somatic mutations (Q62L/E81Q) of AR induced cell clone formation and proliferation function in HCC-LM3 stable expression cell line. The colonies (c-d) and the cell growth function by CCK-8 (e) were shown. Data (mean±SD， *n*=3) was analyzed by one-way ANOVA; **p<0.01;* ***p<0.001;*

****p<0.001. (Scale bar = 1cm)*

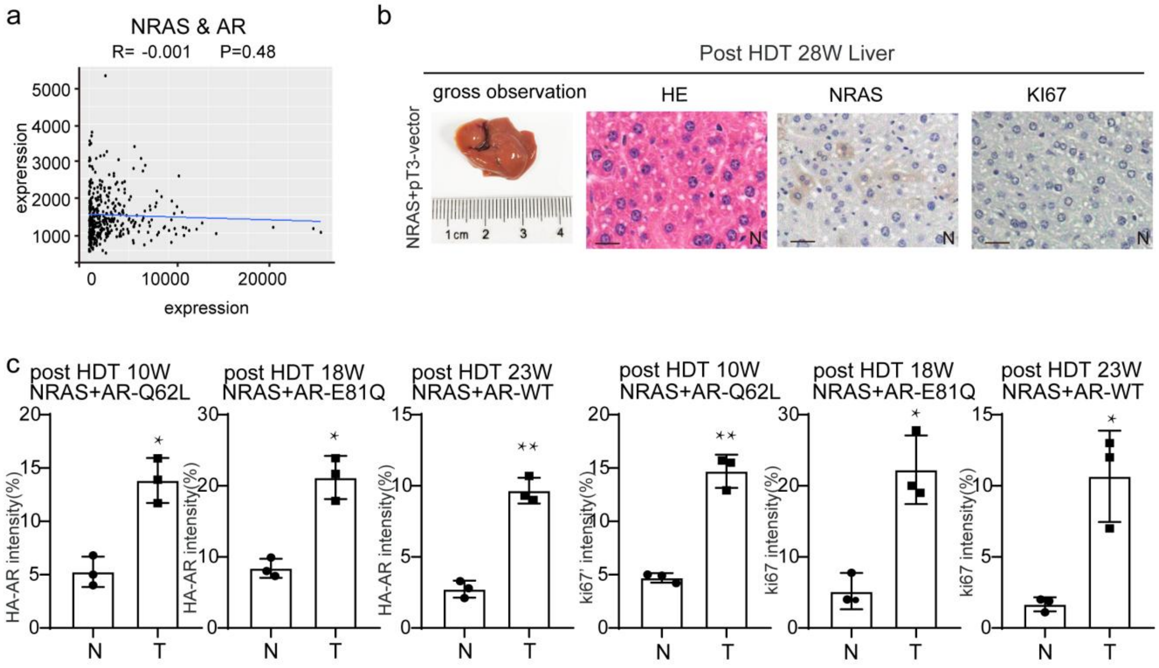

Supplementary Figure 3. NRAS alone didn't induce liver cancer in HDT mice model.

a.The expression levels between AR and NRAS was shown according a TCGA transcriptome dataset consisting of 373 primary HCC tumor samples (http://ualcan.path.uab.edu/). .

b.NRAS/pT3-vector didn't induce liver cancer in HDT mice model. Shown was gross observation and histologic studies of mice liver of NRAS/pT3-vector group at 22W post HDT.*(Scale bar = 50μm)*

c.Quantification of HA-AR and Ki67 expression for IHC staining in Figure 3F by IHC Profile Plugin of ImageJ Software. Data (mean ± SD; n = 3 for each of two animals in each group) were analyzed by unpaired two-tail t-test.N:normal, T:tumor. *$p<0.05$, **$p<0.01$, *** $p < 0.001$.

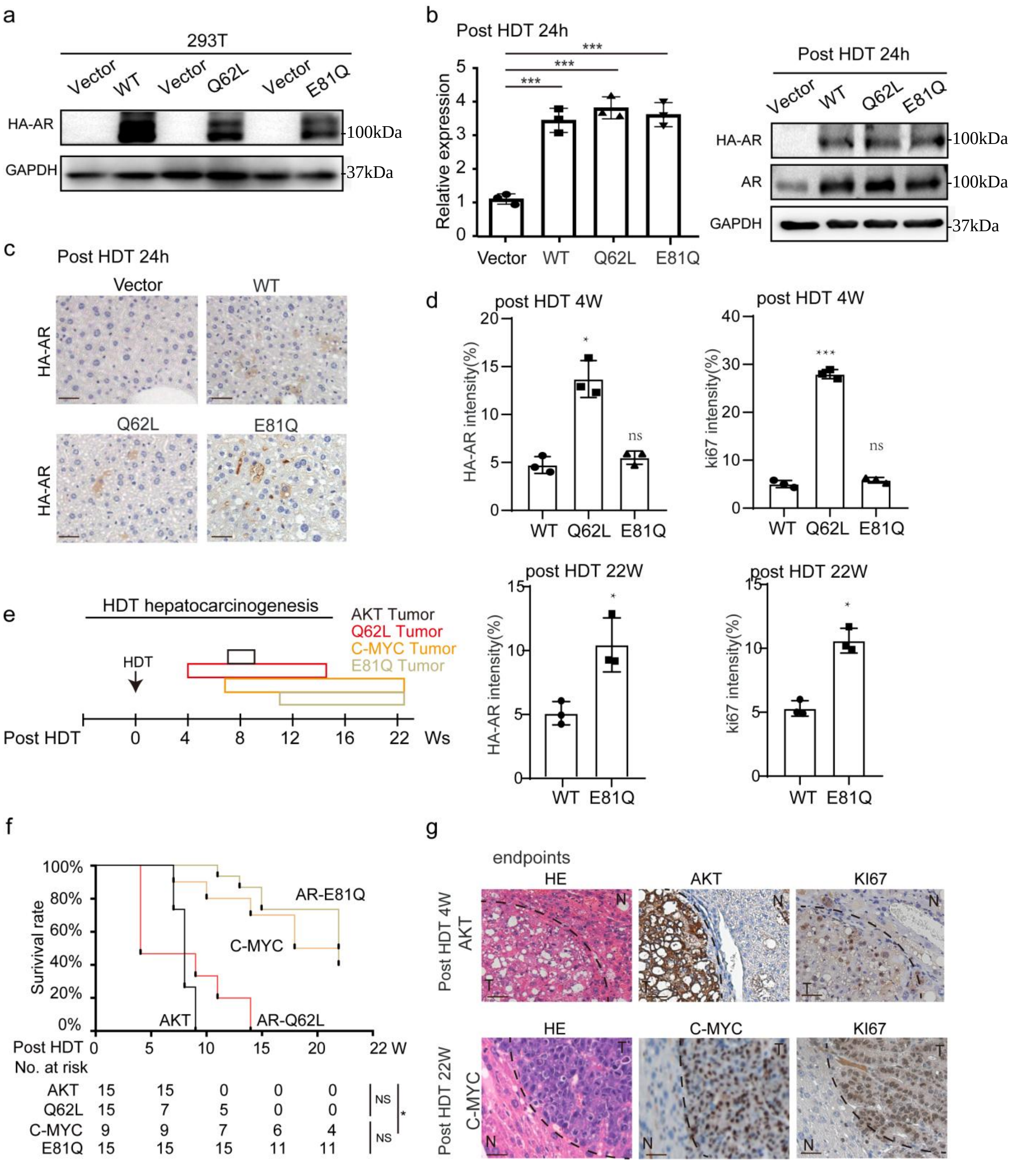

Supplementary Figure 4. The transfection efficiency and malignant degree of AR mutation gene in HDT mice model was shown.

a.The transfection levels of AR mutations in 293Tcells were shown. GAPDH was used as a load. The molecular weights of GAPDH bands were estimated from the manufacturer guidelines.

b.The transfection levels of AR mutations in mice at 24 hours post HDT injection were shown by RT-qPCR against AR and immunoblot with antibody against HA and AR. The mRNA data (Mean ± SD, n = 3) was normalized by GAPDH and analyzed by one-way ANOVA. Protein data was detected by Western Blot and GAPDH was used as a loading.*** p < 0.001.

c.The transfection levels of AR mutations in mice at 24 hours post HDT injection were shown by IHC with antibody against HA *(Scale bar = 50μm).*

d. Quantification of HA-AR and Ki67 expression for IHC staining in Figure 4F-4G by IHC Profile Plugin of ImageJ Software. Data (mean ± SD; n = 3 for each of two animals in each group) were analyzed by one-way ANOVA. *NS:no significance,*p<0.05,**p<0.01, *** p < 0.001.*

e. The diagram of tumor-forming time of AR-mutation-driven compared with other strong oncogene including AKT and C-MYC in HDT mice model.

f. The survival rate and log rank test of AR-mutation-driven and other oncogen-driven HDT mice. *NS:no significance.* *p<0.05.

g. The HE stain of liver cancer of AKT and C-MYC groups were shown *N: normal liver, T: tumor liver (Scale bar = 50μm).*

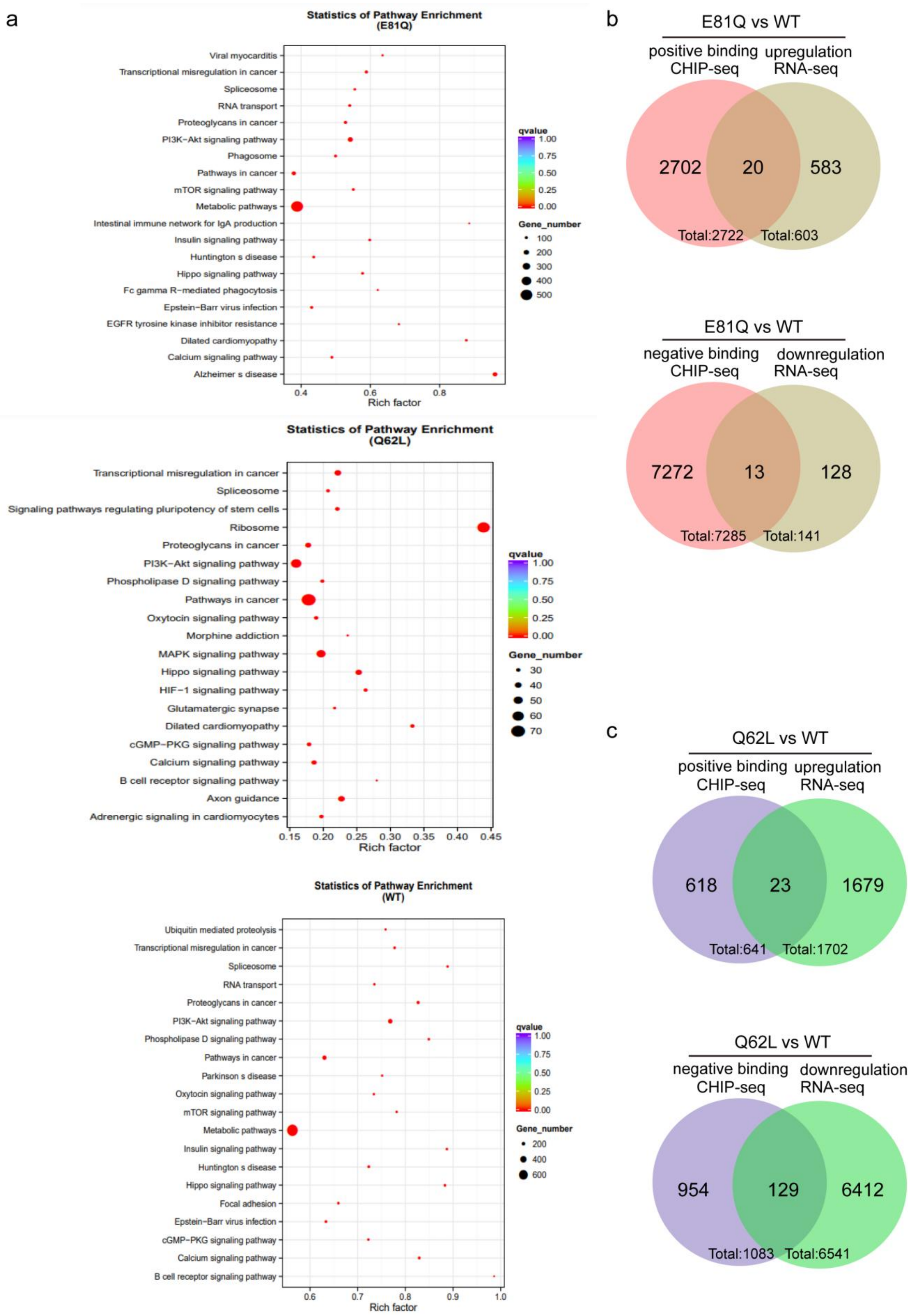

Supplementary Figure 5. The results of RNA-sequencing and ChIP-sequencing.

a.The KEGG analysis of ChIP-Seq data for WT-AR and mutant-AR was presented.

b-c. The transcription (ChIP-Seq) and/or expression (RNA-Seq) regulated genes of AR-Q62L/AR-E81Q compared with AR-WT HDT mice liver tissues were shown.

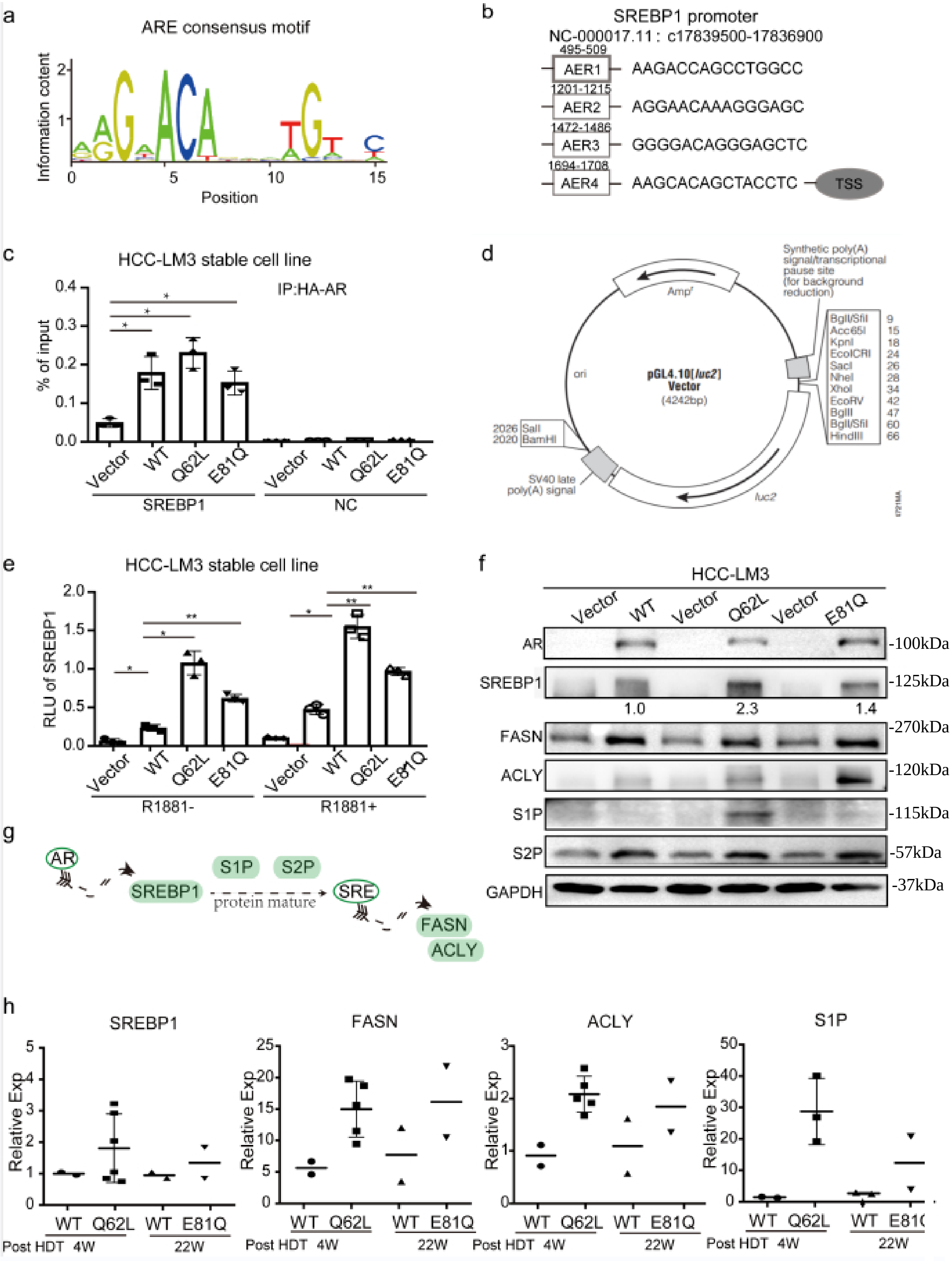

Supplementary Figure 6. AR mutations bond and regulated the transcription of SREBP1 and fat metabolism pathway.

a. AR consensus binding motif based on the JASPAR ChIP-seq database.

(http://jaspar.genereg.net/).

b. Shown were four potential AR binding motifs sequence in SREBP1 promoter.

c. AR bound to SREBP1 promoters. HCC-LM3 cells stably expressing HA-AR mutations or HA-AR WT were assayed for HA-AR binding to SREBP1 promoter by ChIP, which was measured by qPCR. A random sequence was used as a negative control (NC). Data (Mean ± SD， n=3) was analyzed by one-way ANOVA. *NS:no significance, *p<0.05.*

d. Shown were sequencing and structure of pGL4.10 plamids.

e. AR N-term mutations activated the transcription of SREBP. HA-AR WT, HA-AR^{Q62L} and HA-AR^{E81Q} were stably expressed in HCC-LM3 cells carrying SREBP1 promoter-Luc reporter and measured for luciferase activity with or without 10^(-8)M R1881. Data (Mean ± SD, n = 3) was analyzed by one-way ANOVA. **p < 0.05, **p < 0.01,*p<0.001.*

f. The expression level of fat metabolism associated genes (SREBP1/FASN/ACLY/S1P/S2P) were detected by immunoblot in HCC-LM3 stably expressing cells and GAPDH was a loading control.

g, The diagram of AR mutations regulation to SREBP1 dependent fat matabolism.

h.The expression level of fat metabolism associated genes (SREBP1/FASN/ACLY/S1P) were detected by RT-qPCR in different HDT mice. Data (Mean ± SD) was shown and GAPDH was a loading control.

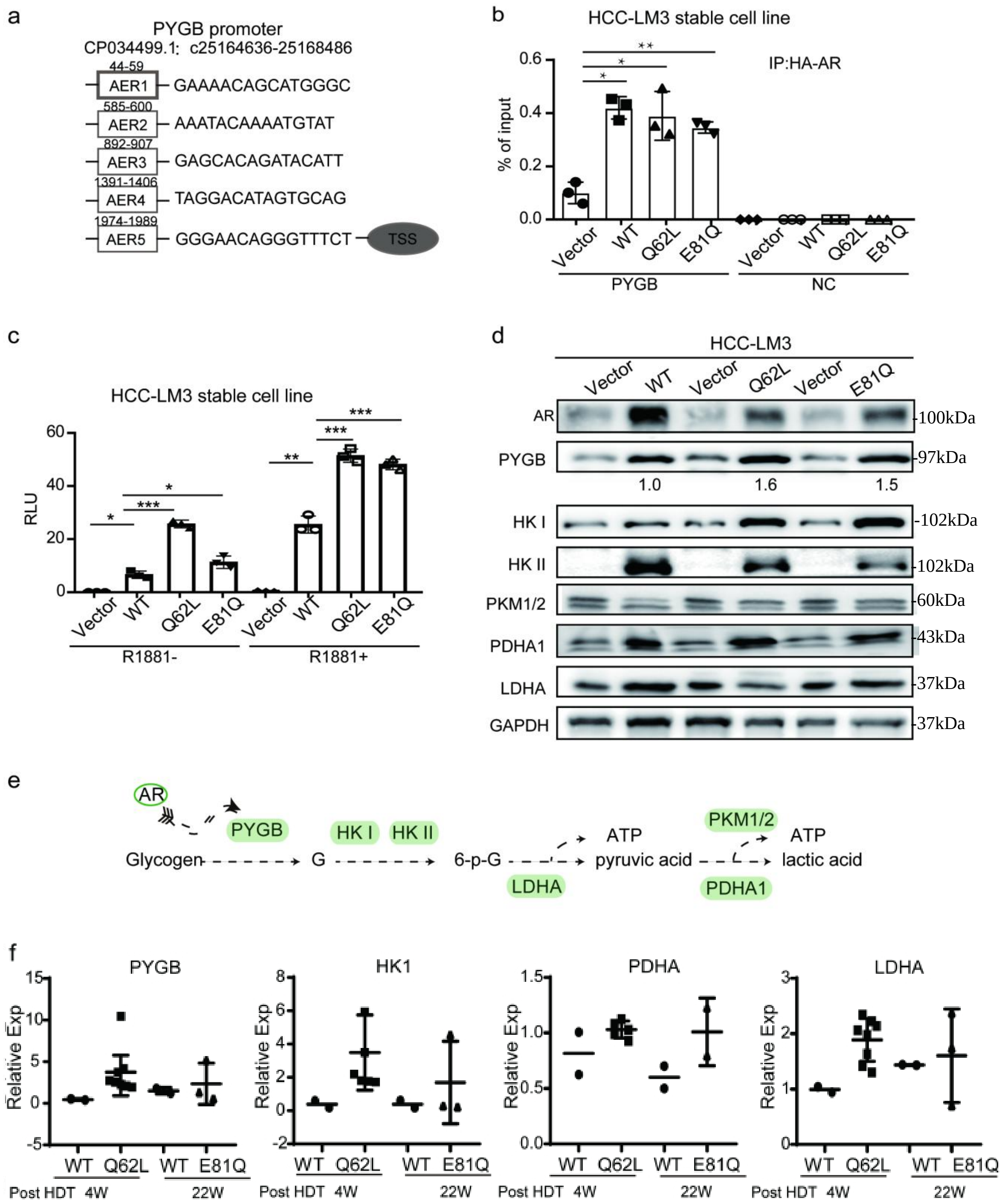

Supplementary Figure 7. AR mutations bound and regulated the transcription of PYGB and glycogen metabolism pathway.

a. Shown were 5 potential AR binding motifs sequence in PYGB promoter.

b. AR bound to PYBG promoters. HCC-LM3 cells stably expressing HA-AR

mutations or HA-AR WT were assayed for HA-AR binding to PYGB promoter by ChIP, which was measured by qPCR. A random sequence was used as a negative control (NC). Data (Mean ± SD， n=3) was analyzed by one-way ANOVA. *$p<0.05$,****$p<0.001$*.

c. AR N-term mutations activated the transcription of PYGB gene. HA-AR WT, HA-AR^{Q62L} and HA-AR^{E81Q} were stably expressed in HCC-LM3 cells carrying PYGB promoter-Luc reporter and measured for luciferase activity with or without 10^(-8)M R1881. Data (Mean ± SD, n = 3) was analyzed by one-way ANOVA. * $p < 0.05$, ** $p < 0.01$,*$p<0.001$.

d. The expression level of glycogen metabolism associated genes (PYGB/HK1/HK2/PKM1/2/PDHA1/LDHA) were detected by immunoblot in HCC-LM3 stably expressing cells and GAPDH was a loading control.

e. The diagram of AR mutations regulation on PYGB associated glycogen metabolism pathway.*G:glucose, 6-P-G: Glucose-6-phosphate.*

f. The expression level of AR mutation mice of glycogen metabolism associated genes were detected by RT-qPCR in different HDT mice. Data (Mean ± SD) was shown and GAPDH was a loading control.

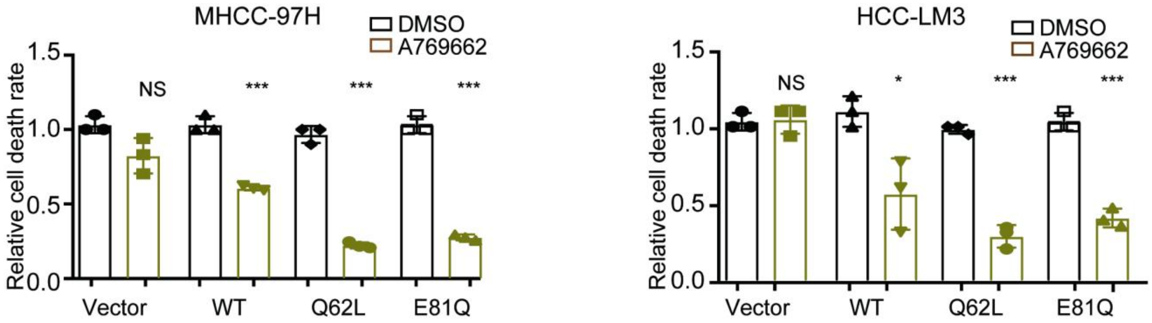

Supplementary Figure 8. A769662 suppressed cell survival of AR mutations expressing hepatoma cells.

The hepatoma cells expressing AR mutations were dealt with 200uM A769662 or DMSO for 48h and the survival rate were analyzed by one-way ANOVA. All error bars represent Standard Deviation.

Supplementary Figure 9. All uncropped Blots.

Shown were all uncropped blots for Figures and Supplementary Figures in the text.

Figure 1d

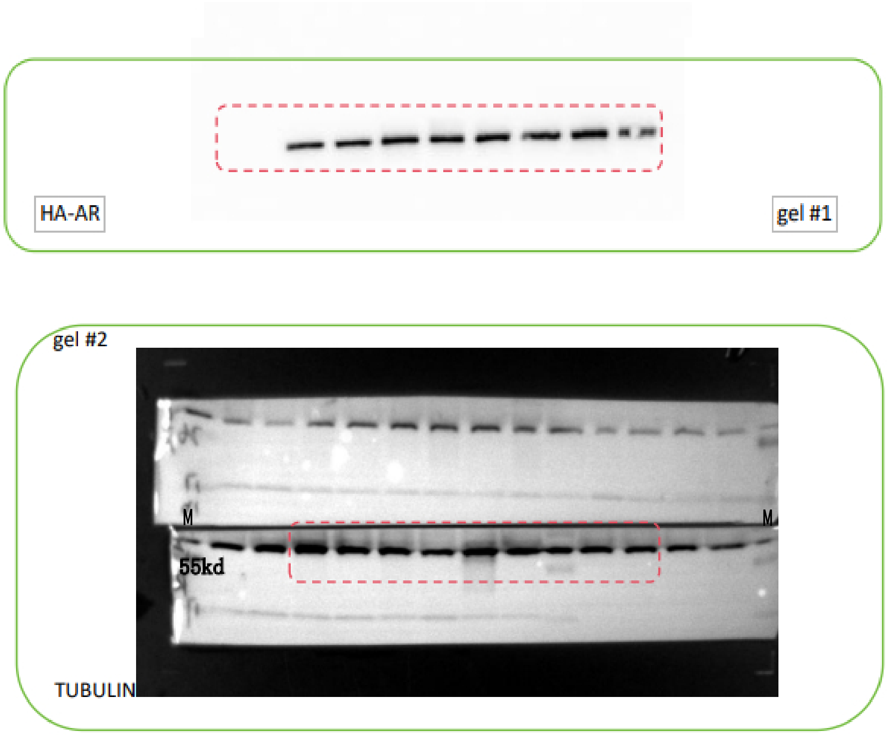

HA-AR were from different lanes on the same gel (gel# 1).
TUBULIN were from different lanes on a separate gel (gel# 2).

Figure 2b

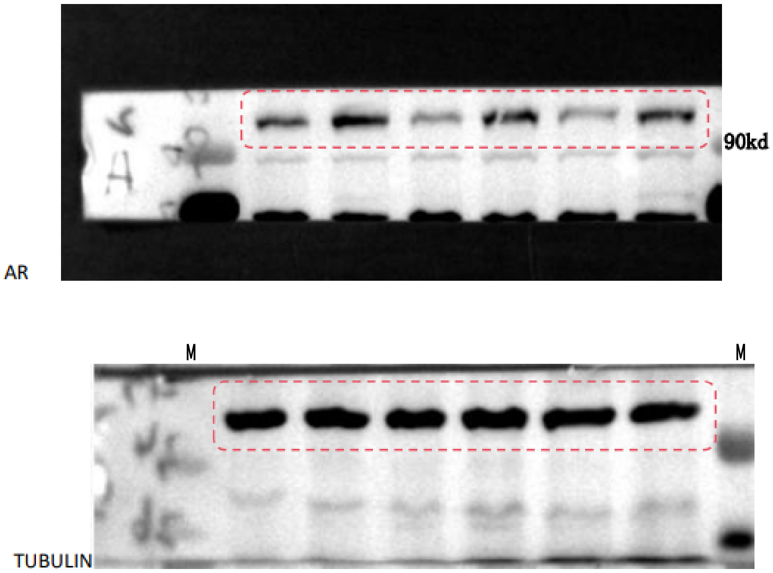

Figure 6d

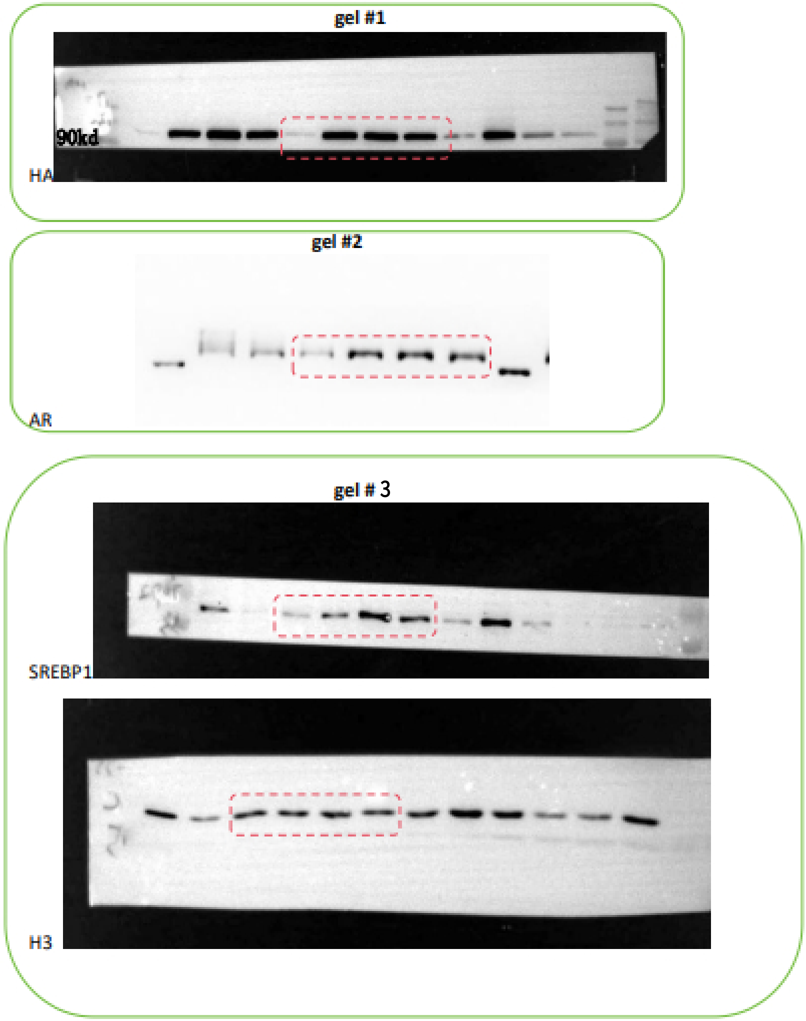

Figure 6f

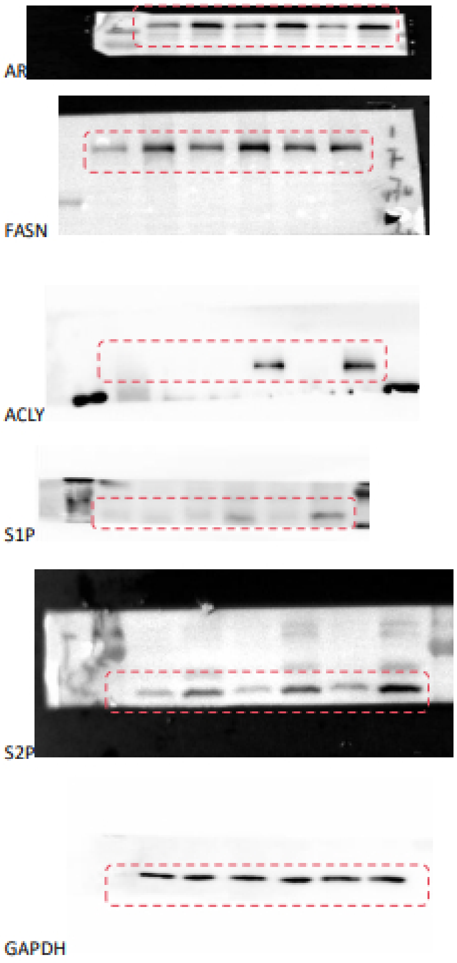

H3, SREBP1 were from different lanes on the same gel (gel# 3).
AR were from different lanes on a separate gel (gel# 2).
HA was run on a separate gel as well (gel# 1).

Figure 6g

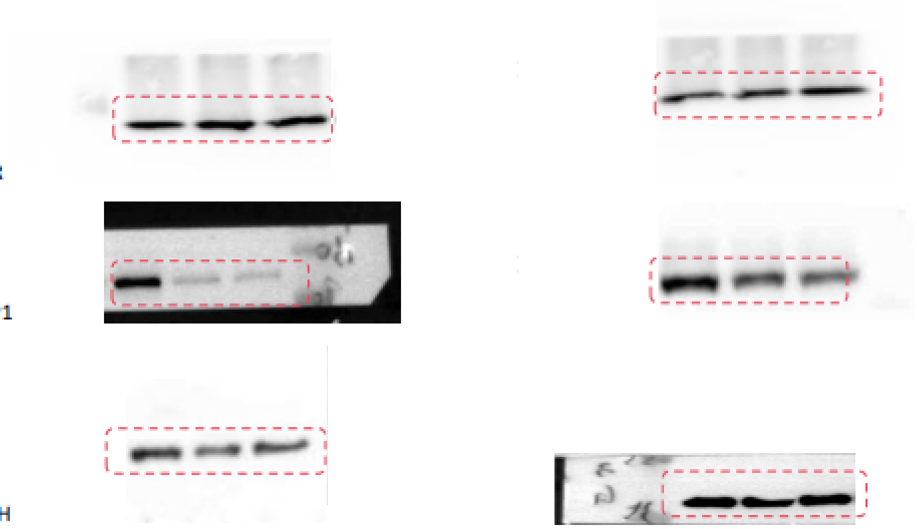

HA-AR

SREBP1

GAPDH

Figure 7d

HA

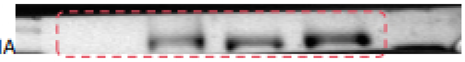

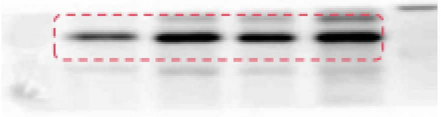

AR

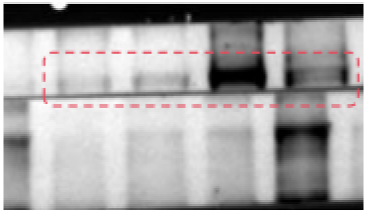

PYGB

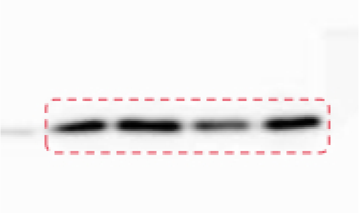

H3

Figure 7f

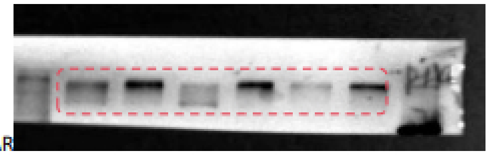

AR

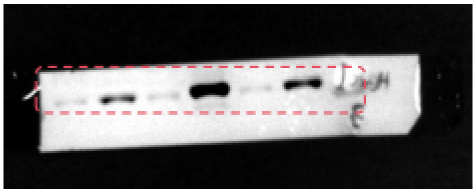

Hk1

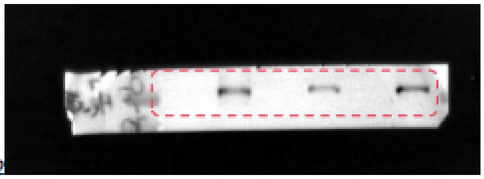

HK2

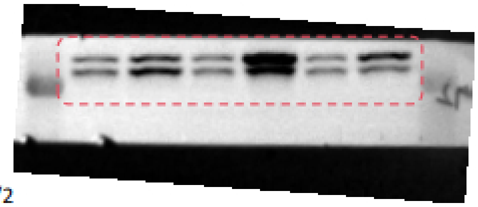

Pkm1/2

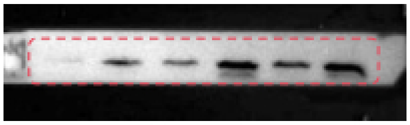

PDHA1

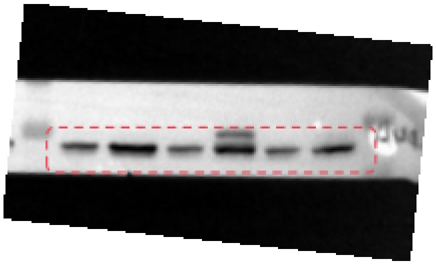

LDHA

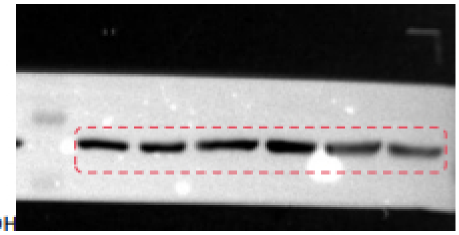

GAPDH

Figure 7g

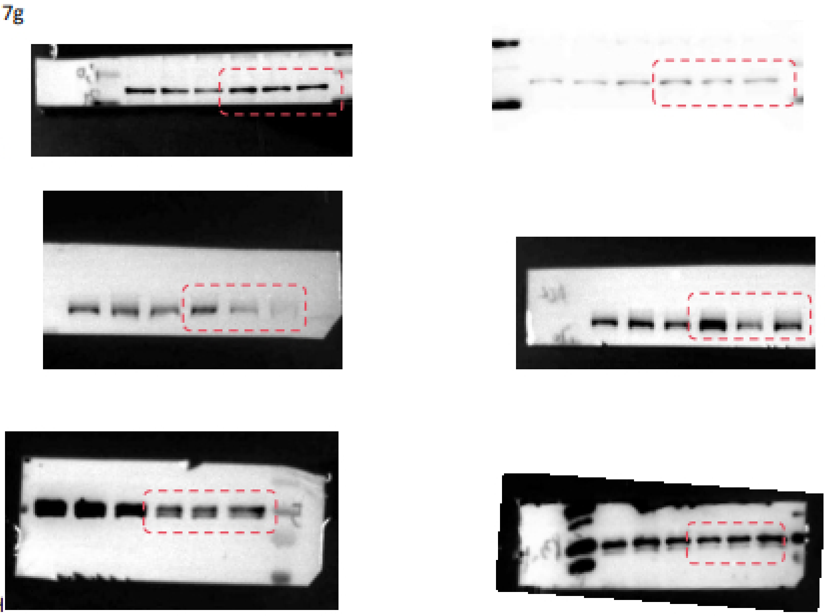

HA-AR

PYGB

GAPDH

Figure 8a

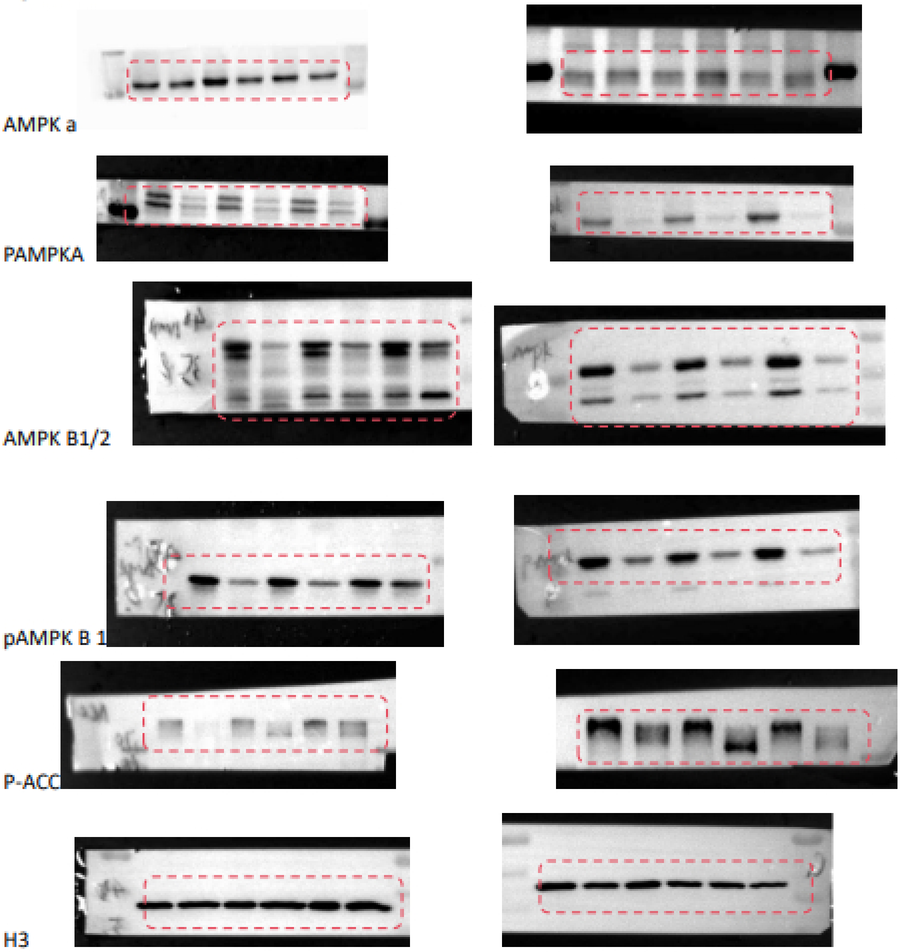

AMPK a

PAMPKA

AMPK B1/2

pAMPK B 1

P-ACC

H3

Figure 8c

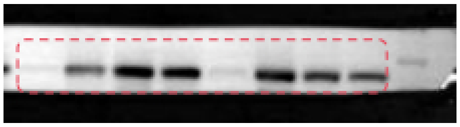

HA

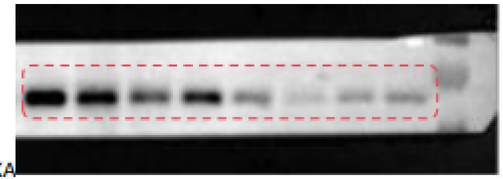

P-AMPKA

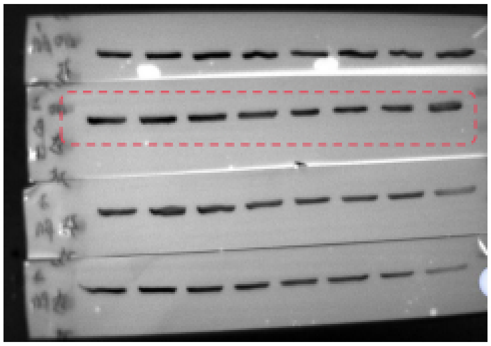

H3

Supplementary figure 2b

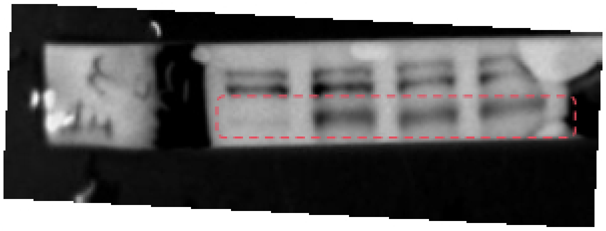

HA-AR

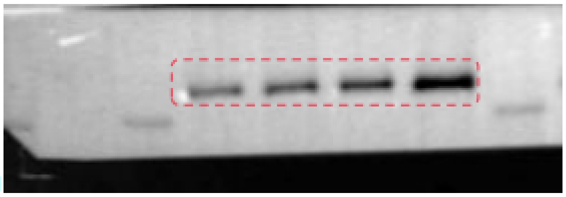

TUBULIN

Supplementary figure 4a

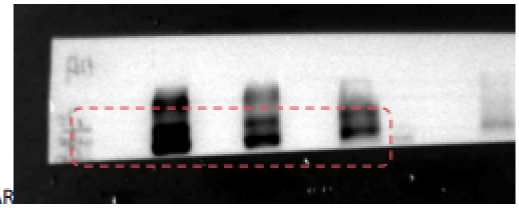

HA-AR

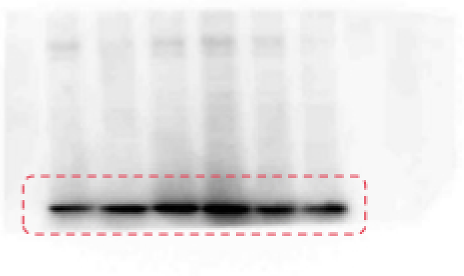

GAPDH

Supplementary figure 4b

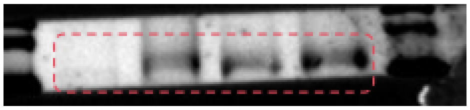

HA-AR

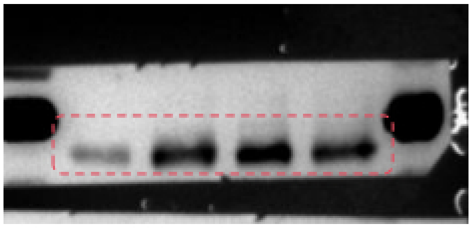

AR

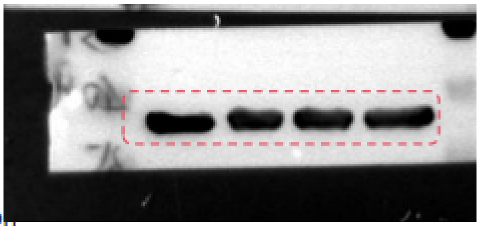

GAPDH

Supplementary figure 6f

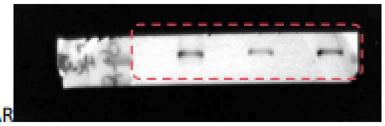

AR

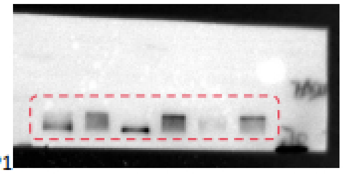

SREBP1

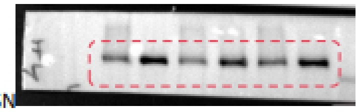

FASN

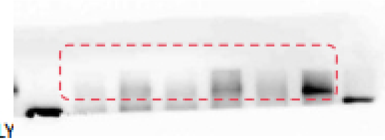

ACLY

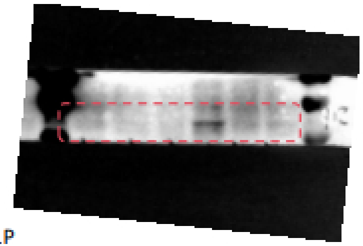

S1P

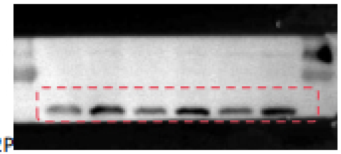

S2P

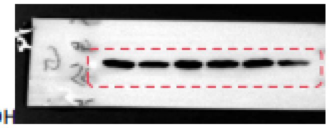

GAPDH

Supplementary figure 7d

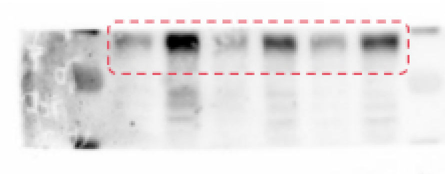

AR

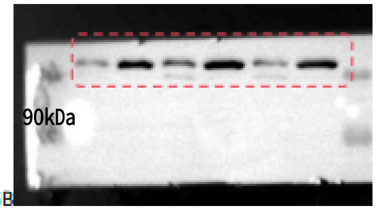

PYGB

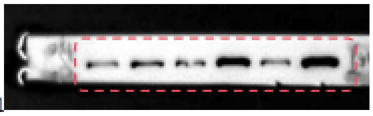

HK1

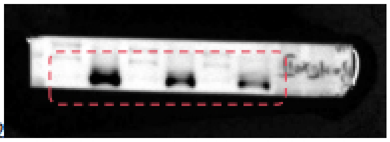

HK2

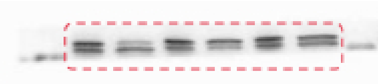

PKM1/2

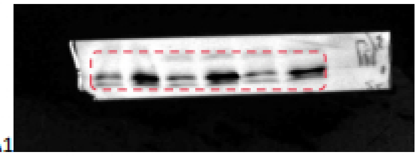

PDHA1

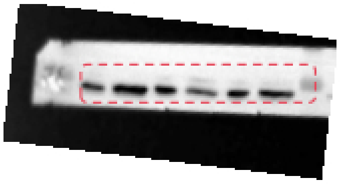

LDHA

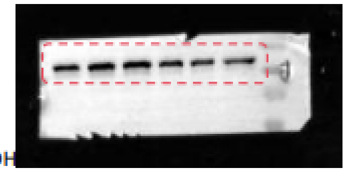

GAPDH

Supplementary Table 1：Antibodies

Antibodies	Brand	Species	Description	Antibodies	Brand	Species	Description
AR	Abcam	Rabbit	Cat#Ab74272	PYGB	Proteintech	Rabbit	Cat#12075-1-AP
HA	CST	Rabbit	Cat#3724	HK1	CST	Rabbit	Cat#2024
TUBLIN	CST	Rabbit	Cat#2148	HK2	CST	Rabbit	Cat#2976
Ki67	proteintech	Rabbit	Cat#27309-1-AP	PFKP	CST	Rabbit	Cat#8164
S1P	Abcam	Rabbit	Cat#Ab59870	PKM1/2	CST	Rabbit	Cat#3190
S2P	Abcam	Rabbit	Cat#Ab244279	PDHA1	CST	Rabbit	Cat#3205
FASN	CST	Rabbit	Cat#3180	LDHA	CST	Rabbit	Cat#3582
SREBP1	Abcam	Rabbit	Cat#28481 AB_778069	H3	CST	Rabbit	Cat#4499
ACLY	Affinity	Rabbit	Cat#BT-0008	NRAS	SAB	Rabbit	Cat#41199
AMPK A	CST	Rabbit	Cat#5831	AMPK B 1/2	CST	Rabbit	Cat#4150
P-AMPK A	CST	Rabbit	Cat#2535	P-AMPK B 1	CST	Rabbit	Cat#4186
c-MYC	abcam	Rabbit	Cat#ab32472	P-ACC	CST	Rabbit	Cat#11818

Supplementary Table 2：qPCR Primers

Forward	Sequence	Reverse	Sequence
**Fasn-F**	GGAGGTGGTGATAGCCGGTAT	**Fasn-R**	TGGGTAATCCATAGAGCCCAG
**Srebp1-F**	TGACCCGGCTATTCCGTGA	**Srebp1-R**	CTGGGCTGAGCAATACAGTTC
**Hk2-F**	TGATCGCCTGCTTATTCACGG	**Hk2-R**	AACCGCCTAGAAATCTCCAGA
**Pfkp-F**	GAAACATGAGGCGTTCTGTGT	**Pfkp-R**	CCCGGCACATTGTTGGAGA
**Scd1-F**	TTCTTGCGATACACTCTGGTG	**Scd1-R**	CGGGATTGAATGTTCTTGTCGT
**Acly-F**	ACCCTTTCACTGGGGATCACA	**Acly-R**	GACAGGGATCAGGATTTCCTTG
**Ldha-F**	TGTCTCCAGCAAAGACTACTG	**Ldha-R**	GACTGTACTTGACAATGTTGGGA
**Pdha-F**	GAAATGTGACCTTCATCGGCT	**Pdha-R**	TGATCCGCCTTTAGCTCCATC
**AR-F**	GGGACATGCGTTTGGAGACT	**AR-R**	TGGGCTGACATTCATAGCCT
**Gapdh-F**	AGGTCGGTGTGAACGGATTTG	**Gapdh-R**	GGGGTCGTTGATGGCAACA
**GAPDH-F**	GGAGCGAGATCCCTCCAAAAT	**GAPDH-F**	GGCTGTTGTCATACTTCTCATGG

Supplementary Table 3：CHIP-qPCR Primers

Name	Forward Primer	Sequence	Reverse Primer	Sequence
**SREBP1 promoter**	Promoter-SREBP1-F	TCACACTTGCAA TCCCAG	Promoter-SREBP1-R	TGGGATTACAGG CGCAC
**PYGB promoter**	Promoter-PYGB-F	CATTCCTTTTAG CAG	Promoter-PYGB-R	ATAAGGGAA ACAGCCAAG
**Negative control**	Irrelevant region-F	AATCTAGCTGAT ATAGTGTGGCTC	Irrelevant region-R	AAGCATACACTT ACACGGCACTCC

Supplementary Table 4：Primers for AR mutagenesis

Forward	Sequence	Reverse	Sequence
**Q62L-F**	GCCAGTTTGCTGCTGCTGCAG CAGCAGCAGCTGCAGCAGCAG CAG	**Q62L-R**	CTGCTGCTGCTGCAGCTGCT GCTGCTGCAGCAGCAGCAA ACTGGC
**E81Q-F**	GCAGCAGCAGCAGCAACAGAC TAGCCCCAGGCAG	**E81Q-R**	CTGCCTGGGGCTAGTCTGTT GCTGCTGCTGCTGC
**A188D-F**	ACATCCTGAGCGAGGACAGCA CCATGCAACT	**A188D-R**	AGTTGCATGGTGCTGTCCTC GCTCAGGATGT
**T440A-F**	CCTGGCACACTCTCTTCGCAGC CGAAGAAGGCC	**T440A-R**	GGCCTTCTTCGGCTGCGAAG AGAGTGTGCCAGG
**G489R-F**	CTCGGCCCCCTCAGCGGCTGGC GGGCC	**G489R-R**	GGCCCGCCAGCCGCTGAGG GGGCCGAG
**C602Y-F**	CCAGCAGAAATGATTACACTAT TGATAAATTCCGA	**C602Y-R**	TCGGAATTTATCAATAGTGT AATCATTTCTGCTGG
**S815N-F**	GCACTGCTACTCTTCAACATTA TTCCAGTGGAT	**S815N-R**	ATCCACTGGAATAATGTTGA AGAGTAGCAGTGC

Supplementary Table 5：Si RNA sequence

Name	Forward Primer
**Si NC**	CCUAAGGUUAAGUCGCCCUCG
**Si PYGB1**	GGUCCUGUAUCCAAAUGAU
**Si PYGB2**	CCCUGUACAAUCGAAUCAA
**Si SREBP1 1**	CGGAGAAGCUGCCUAUCAA
**Si SREBP1 2**	CCACAACGCCAUCGAGAAA

Supplementary Table 6：New Plasmids in Addgene

**Name**	**Addgene ID**
HA-AR-Q62L	213032
HA-AR-E81Q	213033
HA-AR-A188D	213608
HA-AR-T440A	213609
HA-AR-G489R	213610
HA-AR-C602Y	213611
HA-AR-S815N	213612
