# Supplementary material for: Two somatic mutations in the androgen receptor N-terminal domain are oncogenic drivers in hepatocellular carcinoma
Source: Commun Biol. 2024 Jan 5;7:22. doi: 10.1038/s42003-023-05704-2 (PMC10770045; doi:10.1038/s42003-023-05704-2)
Supplement: Supplementary file 2 — Description of Additional Supplementary Files [file 42003_2023_5704_MOESM2_ESM.pdf]

Description of Additional Supplementary Files

File name: Supplementary Data 1

Description: The source data for graphs and charts.
