# Supplementary material for: Two somatic mutations in the androgen receptor N-terminal domain are oncogenic drivers in hepatocellular carcinoma
Source: Commun Biol. 2024 Jan 5;7:22. doi: 10.1038/s42003-023-05704-2 (PMC10770045; doi:10.1038/s42003-023-05704-2)
Supplement: Supplementary file 4 — reporting-summary [file 42003_2023_5704_MOESM4_ESM.pdf]

nature portfolio

Corresponding author(s): QIAN-NAN REN

Last updated by author(s): Nov 18, 2023

Reporting Summary

Nature Portfolio wishes to improve the reproducibility of the work that we publish. This form provides structure for consistency and transparency in reporting. For further information on Nature Portfolio policies, see our Editorial Policies and the Editorial Policy Checklist.

Statistics

For all statistical analyses, confirm that the following items are present in the figure legend, table legend, main text, or Methods section.

n/a	Confirmed	
☐	☒	The exact sample size (n) for each experimental group/condition, given as a discrete number and unit of measurement
☐	☒	A statement on whether measurements were taken from distinct samples or whether the same sample was measured repeatedly
☐	☒	The statistical test(s) used AND whether they are one- or two-sided<br>*Only common tests should be described solely by name; describe more complex techniques in the Methods section.*
☐	☒	A description of all covariates tested
☐	☒	A description of any assumptions or corrections, such as tests of normality and adjustment for multiple comparisons
☐	☒	A full description of the statistical parameters including central tendency (e.g. means) or other basic estimates (e.g. regression coefficient) AND variation (e.g. standard deviation) or associated estimates of uncertainty (e.g. confidence intervals)
☐	☒	For null hypothesis testing, the test statistic (e.g. F, t, r) with confidence intervals, effect sizes, degrees of freedom and P value noted<br>*Give P values as exact values whenever suitable.*
☐	☒	For Bayesian analysis, information on the choice of priors and Markov chain Monte Carlo settings
☐	☒	For hierarchical and complex designs, identification of the appropriate level for tests and full reporting of outcomes
☐	☒	Estimates of effect sizes (e.g. Cohen's d, Pearson's r), indicating how they were calculated

Our web collection on statistics for biologists contains articles on many of the points above.

Software and code

Policy information about availability of computer code

Data collection	No software or code was used in this study
Data analysis	No software or code was used in this study

For manuscripts utilizing custom algorithms or software that are central to the research but not yet described in published literature, software must be made available to editors and reviewers. We strongly encourage code deposition in a community repository (e.g. GitHub). See the Nature Portfolio guidelines for submitting code & software for further information.

Data

Policy information about availability of data

All manuscripts must include a data availability statement. This statement should provide the following information, where applicable:

- Accession codes, unique identifiers, or web links for publicly available datasets
- A description of any restrictions on data availability
- For clinical datasets or third party data, please ensure that the statement adheres to our policy

Yes. this manuscript includes a data availability statement.

Research involving human participants, their data, or biological material

Policy information about studies with human participants or human data. See also policy information about sex, gender (identity/presentation), and sexual orientation and race, ethnicity and racism.

Reporting on sex and gender	No
Reporting on race, ethnicity, or other socially relevant groupings	No
Population characteristics	No
Recruitment	No
Ethics oversight	No

Note that full information on the approval of the study protocol must also be provided in the manuscript.

Field-specific reporting

Please select the one below that is the best fit for your research. If you are not sure, read the appropriate sections before making your selection.

☒ Life sciences ☐ Behavioural & social sciences ☐ Ecological, evolutionary & environmental sciences

For a reference copy of the document with all sections, see nature.com/documents/nr-reporting-summary-flat.pdf

Life sciences study design

All studies must disclose on these points even when the disclosure is negative.

Sample size	Animal experiments including 5-15 mice for each groups.
Data exclusions	No data from this study were excluded from analysis. Our exclusion criteria were: the HD transgenic animals were not included in the analysis if they were not successfully constructed
Replication	Each experiment was independently repeated or set with relatively large samples for validation.
Randomization	All the animals involved in this research were randomized.
Blinding	Double blinding was not required for our animal experiments.

Reporting for specific materials, systems and methods

We require information from authors about some types of materials, experimental systems and methods used in many studies. Here, indicate whether each material, system or method listed is relevant to your study. If you are not sure if a list item applies to your research, read the appropriate section before selecting a response.

Materials & experimental systems

n/a	Involved in the study	
☐	☒	Antibodies
☐	☒	Eukaryotic cell lines
☒	☐	Palaeontology and archaeology
☐	☒	Animals and other organisms
☒	☐	Clinical data
☒	☐	Dual use research of concern
☒	☐	Plants

Methods

n/a	Involved in the study	
☐	☒	ChIP-seq
☒	☐	Flow cytometry
☒	☐	MRI-based neuroimaging

Antibodies

Antibodies used	see in Key resource tables
Validation	see in Key resource tables

Eukaryotic cell lines

Policy information about cell lines and Sex and Gender in Research

Cell line source(s)	see in methods
Authentication	see in methods
Mycoplasma contamination	see in methods
Commonly misidentified lines (See ICLAC register)	No

Animals and other research organisms

Policy information about studies involving animals; ARRIVE guidelines recommended for reporting animal research, and Sex and Gender in Research

Laboratory animals	FVB/N mice
Wild animals	NO
Reporting on sex	male mice
Field-collected samples	No
Ethics oversight	Yes, see in methods

Note that full information on the approval of the study protocol must also be provided in the manuscript.

Plants

Seed stocks	not used
Novel plant genotypes	not used
Authentication	not used

ChIP-seq

Data deposition

☒ Confirm that both raw and final processed data have been deposited in a public database such as GEO.

☒ Confirm that you have deposited or provided access to graph files (e.g. BED files) for the called peaks.

Data access links *May remain private before publication.*	https://www.ncbi.nlm.nih.gov/geo/query/acc.cgi?acc=GSE249686
Files in database submission	CHIP-seq
Genome browser session (e.g. UCSC)	All data was shown in database.

Methodology

Replicates	3
Sequencing depth	All data was shown in database.
Antibodies	AR antibodies
Peak calling parameters	All data was shown in database.

Data quality	All data was shown in database.
Software	All data was shown in database.
